# Insights into the Mechanism of Bovine CD38/NAD+Glycohydrolase from the X-Ray Structures of Its Michaelis Complex and Covalently-Trapped Intermediates

**DOI:** 10.1371/journal.pone.0034918

**Published:** 2012-04-18

**Authors:** Pascal F. Egea, Hélène Muller-Steffner, Isabelle Kuhn, Céline Cakir-Kiefer, Norman J. Oppenheimer, Robert M. Stroud, Esther Kellenberger, Francis Schuber

**Affiliations:** 1 Department of Biological Chemistry, University of California Los Angeles, Los Angeles, California, United States of America; 2 Laboratoire de Conception et Application de Molécules Bioactives, UMR 7199 CNRS, Université de Strasbourg, Faculté de Pharmacie, Illkirch, France; 3 Unité de Recherche Animal et Fonctionnalités des Produits Animaux, UR AFPA, Nancy Université, Vandoeuvre-les-Nancy, France; 4 Department of Pharmaceutical Chemistry, University of California San Francisco, San Francisco, California, United States of America; 5 Department of Biochemistry and Biophysics, University of California San Francisco, San Francisco, California, United States of America; 6 Laboratoire d'Innovation Thérapeutique, UMR 7200 CNRS, Université de Strasbourg, Faculté de Pharmacie, Illkirch, France; Institute of Enzymology of the Hungarian Academy of Science, Hungary

## Abstract

Bovine CD38/NAD^+^glycohydrolase (bCD38) catalyses the hydrolysis of NAD^+^ into nicotinamide and ADP-ribose and the formation of cyclic ADP-ribose (cADPR). We solved the crystal structures of the mono *N*-glycosylated forms of the ecto-domain of bCD38 or the catalytic residue mutant Glu218Gln in their *apo* state or bound to aFNAD or rFNAD, two 2′-fluorinated analogs of NAD^+^. Both compounds behave as mechanism-based inhibitors, allowing the trapping of a reaction intermediate covalently linked to Glu218. Compared to the non-covalent (Michaelis) complex, the ligands adopt a more folded conformation in the covalent complexes. Altogether these crystallographic snapshots along the reaction pathway reveal the drastic conformational rearrangements undergone by the ligand during catalysis with the repositioning of its adenine ring from a solvent-exposed position stacked against Trp168 to a more buried position stacked against Trp181. This adenine flipping between conserved tryptophans is a prerequisite for the proper positioning of the N1 of the adenine ring to perform the nucleophilic attack on the C1′ of the ribofuranoside ring ultimately yielding cADPR. In all structures, however, the adenine ring adopts the most thermodynamically favorable *anti* conformation, explaining why cyclization, which requires a *syn* conformation, remains a rare alternate event in the reactions catalyzed by bCD38 (cADPR represents only 1% of the reaction products). In the Michaelis complex, the substrate is bound in a constrained conformation; the enzyme uses this ground-state destabilization, in addition to a hydrophobic environment and desolvation of the nicotinamide-ribosyl bond, to destabilize the scissile bond leading to the formation of a ribooxocarbenium ion intermediate. The Glu218 side chain stabilizes this reaction intermediate and plays another important role during catalysis by polarizing the 2′-OH of the substrate NAD^+^. Based on our structural analysis and data on active site mutants, we propose a detailed analysis of the catalytic mechanism.

## Introduction

Mammalian NAD^+^glycohydrolases (NADases; EC 3.3.2.5 and 3.2.2.6) catalyze the hydrolytic cleavage of the nicotinamide-ribose bond of NAD(P)^+^. Most of them also catalyze base-exchange (transglycosidation) reactions giving access to pyridinium analogs of NAD(P)^+^. For many decades NADases were considered to form a rather heterogeneous group of enzymes, in terms of apparent molecular weights and catalytic properties, that are widely distributed in many organisms [1]. The kinetic and molecular mechanisms of these enzymes have been extensively studied [1], [2] and, in this respect, bovine spleen NAD^+^glycohydrolase represents an archetypal NADase we have thoroughly investigated over the years [3].

In sharp contrast with the results derived from studying the molecular enzymology of NADases, knowledge of their structure and the deciphering of their biological function(s) remained limited. Although preponderantly described as ecto-enzymes [4], some NADases were also found in intracellular compartments of various tissues/cells and a role in NAD^+^ salvage pathways was thus tentatively ascribed to this class of enzymes. This situation experienced an unexpected paradigmic shift with the discovery, in invertebrates, by the group of H.C. Lee, of cyclic ADP-ribose (cADPR), a new calcium mobilizing messenger [5], and of ADP-ribosyl cyclase, a soluble enzyme able to convert NAD^+^ quasi-exclusively into this cyclic metabolite [6]. Determination of the structure of the cyclase from *Aplysia californica* revealed its striking structural similarity with human CD38 (hCD38), a 46-kDa type II transmembrane glycoprotein known as a surface antigen of lymphoid cells of unknown biochemical functions [7]. It was then established that mammalian CD38 were indeed enzymes overwhelmingly endowed with NAD^+^glycohydrolase activity. In addition, CD38 is also able to catalyze the conversion of NAD^+^ to cADPR, albeit only with very low yields [8], and the hydrolysis of cADPR to ADP-ribose (ADPR) [9]. GPI-anchored CD157 represents an additional member of the CD38/ADP-ribosyl cyclase gene family. Its catalytic functions which, compared to NADases and CD38 were less explored, are quite similar although characterized by a much lower efficiency [10]. Simplifying our perception of these different enzymes, our group has subsequently shown that the much studied ‘classical’ bovine NADase was also able to catalyze, like CD38, ADP-ribosyl cyclase (<2% of reaction products) and cyclic ADP-ribose hydrolase reactions [11], [12]. After partial peptide sequence determination and molecular cloning, the 32-kDa bovine NAD^+^glycohydrolase was finally identified as a member of the CD38 family [13]. Thus, the world of the classical mammalian NAD^+^glycohydrolases merged with that of CD38 [14].

After cADPR, NAADP^+^ whose biosynthesis was ascribed to a base-exchange reaction between NADP^+^ and nicotinic acid catalyzed by CD38, also joined the ranks of potent calcium mobilizing metabolites [15]. Finally ADP-ribose, the main reaction product of CD38 was also shown to regulate calcium-permeable TRPM2 channels [16]. Altogether CD38 appears to be a key player in the biosynthesis of calcium messengers which are involved in a wide range of cellular functions [17]. Since the catalytic activity of CD38 is also related to diseases, such as diabetes, asthma, and inflammation, this enzyme is an interesting pharmacological target.

**Figure 1:**
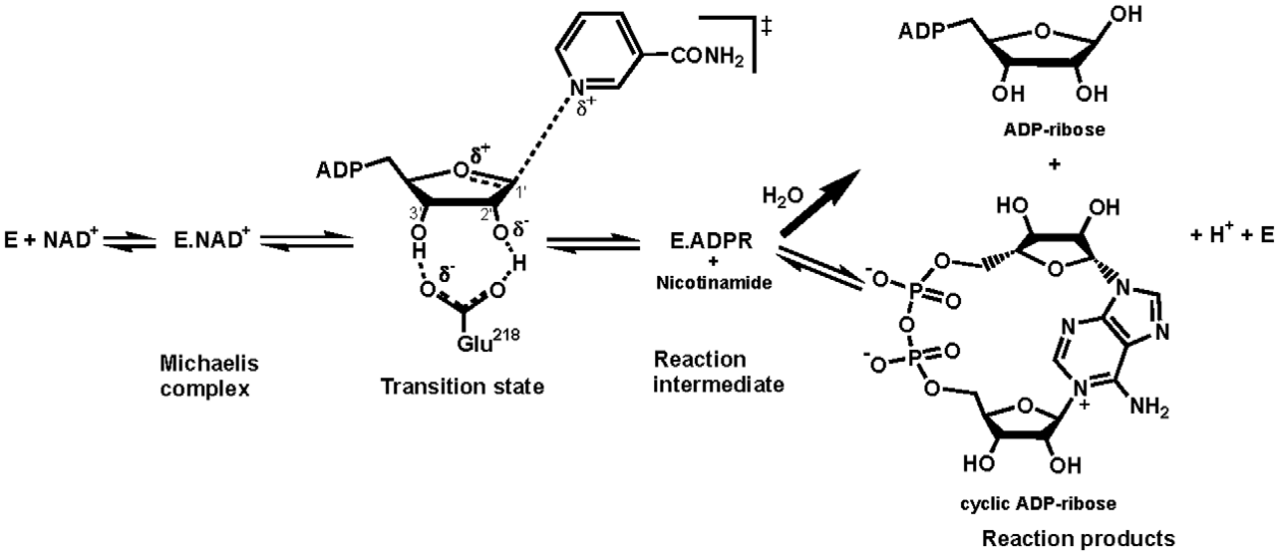
Partitioning reaction mechanism of bCD38. The nicotinamide-ribosyl bond of NAD^+^ is cleaved via a dissociative process with a late transition state, leading to a ribooxocarbenium ion reaction intermediate stabilized by the side-chain of invariant Glu218. This rate-determining step is followed by two nucleophilic reactions in competition: (i) an *intermolecular* pathway involving a rapid trapping from the β-face of this intermediate by a water molecule (NAD^+^ glycohydrolase activity) or by competing neutral nucleophiles such as pyridines (transglycosidation reactions) or alcohols (e.g., methanolysis), and (ii) an *intramolecular* reaction between N1 of the adenine ring and C1′ (anomeric carbon) of the oxocarbenium ion leading to the formation of cyclic ADP-ribose (ADP-ribosyl cyclase activity). This latter reaction represents a kinetically minor step (∼1% reaction products) relative to solvolysis.

The observed ‘multifunctionality’ of CD38 prompted a reinvestigation of the mechanism of bovine CD38/NAD^+^glycohydrolase (bCD38). We have demonstrated the occurrence of an unifying partitioning mechanism that applies to all members of the CD38/ADP-ribosyl cyclase enzyme family [12], [18], which was borne out by other groups [19]. Thus, the catalytic transformation of NAD^+^ involves the formation, via a ribooxocarbenium ion-like transition state (Figure 1), of a unique reaction intermediate that partitions between several competing pathways such as an irreversible hydrolytic process leading to ADP-ribose and a reversible minor pathway that governs the formation of cADPR [3].

Bovine CD38/NAD^+^glycohydrolase presents some distinct structural features amongst mammalian CD38. Its ecto-domain shares about 48% sequence identity with the ecto-domain of hCD38. It is missing seven residues at the C-terminus and an otherwise highly conserved disulfide bond [13]. Furthermore, bCD38 contains only two *N*-glycosylation motifs, of which just one is occupied at Asn201 [20], instead of the four observed in all others CD38. The impact of these differences on the structure and the mechanism of bCD38 is unknown.

In this extensive crystallographic study we report five high resolution (1.55–1.95 Å) structures of the mono *N*-glycosylated ecto-domain of wild-type (wt) bCD38 and a catalytic residue mutant (E218Q) in *apo*, non-covalent and covalent complexes, captured with 2′-fluorinated analogs of NAD^+^ acting as mechanism-based inhibitors. Besides affording the structure of a novel CD38 distinct from the human one [21], our main aim was to elucidate mechanistic aspects of this enzyme at a molecular level such as: i) the destabilization of the nicotinamide-ribose bond in NAD^+^ which has represented a puzzle to enzymologists for many years, ii) the reaction intermediate stabilization, and iii) the origin of the low percentage of transformation of NAD^+^ into cADPR. Our structures survey and sample different enzyme-substrate conformational and reactive states along the catalytic pathways mediated by CD38. These crystallographic snapshots reveal the drastic conformational rearrangements undergone by the substrate within the catalytic site. Our structural investigations of bCD38 provide clear insights into the modalities of substrate binding that are relevant to the scissile bond destabilization and to the cyclization process. Based on our structural analysis combined to enzymatic measurements on active site mutants, we propose a detailed analysis of the catalytic mechanism for this enzyme.

## Results and Discussion

### Architecture of bovine CD38

As reported previously, we have expressed in *Pichia pastoris* a recombinant form of bovine CD38/NAD^+^glycohydrolase truncated for the first 31 amino acids that encompass the transmembrane and short intracellular domains [20]. The construct used for crystallization consists in this soluble ecto-domain (residues 32–278) of bCD38 [13]. In contrast with the other known mammalian CD38, such as hCD38 which contains four *N*-glycosylation sites [7], bCD38 is a mono-glycosylated protein at position Asn201 and its only other sequon at Asn268 is unoccupied [20]. The glycosylation of bCD38 did not prevent crystallization or adversely affect the diffraction quality of crystals. The 1.8 Å structure of *apo* bCD38 was solved by molecular replacement using the known hCD38 structure (PDB code 1yh3) as model [21]. The invariant glutamate 218 identified as the catalytic residue of bCD38 (see below) was mutated into glutamine (E218Q); the structure of this mutant was solved at high resolution (1.55 Å) (Table 1 and Material and Methods).

**Table 1:** X-ray diffraction data and structure refinement statistics for wild-type bCD38 and E218Q mutant structures.

Structure	wt bCD38	wt bCD38 covalent complex with aFNAD	wt bCD38 covalent complex with rFNAD	mutant bCD38	mutant bCD38 NON-covalent complex with rFNAD
PDB ID	3GH3	3P5S	3KOU	3GC6	3GHH
Data set statistics	ALS100606	ALS250407	ALS060507	ALS250407	ALS060507
Wavelength (Å)	1.11587	1.11587	1.11587	1.11587	1.11587
Space group	P2_1_2_1_2_1_	P2_1_2_1_2_1_	P2_1_2_1_2_1_	P2_1_2_1_2_1_	P2_1_2_1_2_1_
Cell dimensions					
*a* (Å)	47.4	46.2	46.7	47.0	46.4
*b* (Å)	80.9	80.0	81.3	80.2	79.4
*c* (Å)	151.8	157.7	150.9	152.5	156.3
Resolution (Å)	50.0–1.80	80.2–1.95	75.4–1.78	80.1–1.55	50.0–1.94
(last shell) (Å)	(1.86–1.80)	(2.06–1.95)	(1.88–1.78)	(1.61–1.55)	(2.01–1.94)
Unique reflections	53,100 (4,812)	39,086 (5,881)	52,041 (6,599)	74,734 (4,271)	41,975 (4,109)
Redundancy	3.9 (3.4)	2.9 (2.7)	3.5 (3.3)	3.3 (1.3)	3.4 (3.1)
Completeness (%)	97 (89)	90 (94)	93 (82)	82 (33)	96 (96)
*I/σ(I)*	26.0 (2.9)	7.1 (2.1)	8.0 (1.5)	16.9 (1.5)	11.2 (2.0)
*R_sym_* (%)	3.4 (30.6)	8.4 (39.1)	9.9 (77.5)	3.5 (31.8)	7.2 (61.0)
Refinement statistics					
Resolution range (Å)	40.4–1.80	78.8–1.95	75.4–1.78	76.2–1.55	78.1–1.94
Reflections used work (test)	47,191 (3,873)	34,641 (2,902)	47,602 (4,018)	63,095 (5,756)	38,617 (3,242)
*R_free_* (%)/*R_fac_* (%)	22.3/17.7	25.6/20.5	24.3/20.0	21.0/18.7	25.4/20.6
Overall figure of merit	0.932	0.890	0.920	0.950	0.917
Overall *B_wilson_* (Å^2^)	18	26	22	21	28
Protein atoms, *B* (Å^2^)	3,892, 29	3,811, 33	3,798, 28	4,022, 39	3,855, 38
Ligand atoms (Å^2^)	no ligand	70, 37	52, 29	no ligand	41, 47
Solvent atoms, *B* (Å^2^)	528, 39	355, 36	368, 33	488, 49	280, 42
Carbohydrate atoms, *B* (Å^2^)	56, 62	28, 58	42, 42	56, 65	56, 59
Ions, *B* (Å^2^)	2 sulfates, 90	2 sulfates, 48	2 sulfates, 58	5 sulfates, 65	4 sulfates, 61
	1 cacodylate, 78				
rmsd bonds (Å)^a^	0.008	0.007	0.011	0.011	0.006
rmsd angles (°)	1.124	1.226	1.204	1.265	1.098
Ramachandran analysis					
Preferred regions (%)	95.9	95.8	92.9	95.9	92.0
Allowed regions (%)	3.9	3.8	6.0	3.9	5.4
Outliers (%)	0.2	0.4	1.1	0.2	2.6

**Figure 2:**
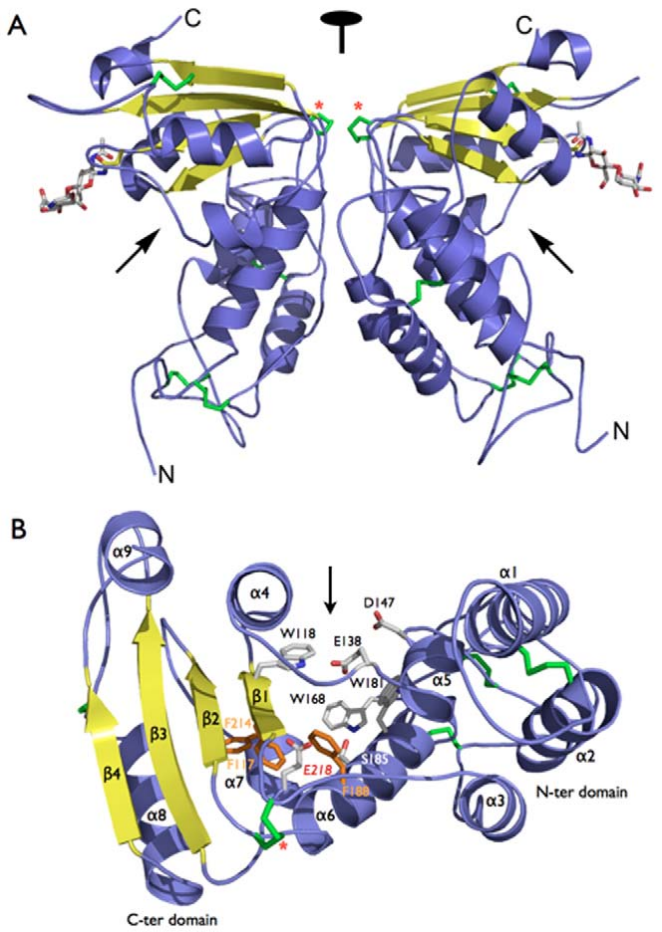
Overview of the structure of bCD38 and its active site. (A) Structure of the homodimer of the ecto-domain (Δ31) of wt *apo* bCD38 observed in the asymmetric unit; the non-crystallographic two-fold axis is indicated. (B) The active site of bCD38. The side chains of selected residues participating to the catalytic activity of the enzyme are indicated in grey. The phenylalanine ‘plug’ is in orange. Catalytic residue Glu218 sits at the bottom of the active site pocket. Secondary structure elements are labeled. The N- and C- terminal domains are indicated. The five disulfide bonds stabilizing each monomer are depicted in green and a red asterisk indicates the disulfide bond specific to CD38 that stabilizes its hinge region. The sugar chains bound to residues Asn201 are shown in stick representation. The active sites are indicated with arrows.

The L-shaped structure of bCD38 consists of two distinct domains separated by a cleft and connected by a hinge region (Figure 2A). α-Helices are found mainly in the N-terminal and membrane-proximal domain of this transmembrane protein, whereas β-sheets are preponderant in the distal C-terminal domain. The N-ter domain (residues 41–110 and 137–191) is a bundle of five α-helices (α1, α2, α3, α5 and α6) and the C-ter domain (residues 113–134 and 194–278) consists of a four-stranded parallel β-sheets (β1, β2, β3 and β4) surrounded by two long (α4 and α8) and two shorter and more dynamic α-helices (α7 and α9). Residues 111–112, 135–136, and 192–193 constitute the hinge region. As predicted [13], bCD38 is stabilized by four disulfide bonds, Cys59–Cys75, Cys92–Cys172, Cys152–Cys165 and Cys244–Cys265, which are conserved in all members of the CD38/ADP-ribosyl cyclase family. The bovine C-terminal domain is naturally shorter by 12 amino acids compared to the other CD38, and is thus missing their fifth and otherwise conserved disulfide bond (Cys287–Cys296 in hCD38) with no consequences on its catalytic activity. However, like hCD38, a supplementary disulfide bond absent in invertebrate ADP-ribosyl cyclases and which seems unique to CD38, was also found between Cys112 and Cys193 of bCD38, stabilizing its hinge region (Figure 2A). We have shown previously [13], that the N-terminal α1 helix is dispensable for the conformational stability of the bovine enzyme and for its catalytic activity (Figure S1). The overall architecture of the glycosylated form of bCD38/NAD^+^glycohydrolase shows a great resemblance to its hCD38 homolog, which has 39% sequence identity and whose four *N*-glycosylation sites were silenced by mutagenesis [21]. Superposition of the E218Q mutant on the wt *apo* enzyme showed no differences between the structures (not shown) demonstrating the conservative nature of the mutation. This suggests that the active site geometry is maintained in the mutant and that the kinetic effects of the mutation (see below) are due to an intrinsic catalytic function of Glu218 side chain.

### bCD38 associates as a parallel ‘back-to-back’ homodimer

All crystals of wt bCD38 and mutant belong to the orthorhombic space group P2_1_2_1_2_1_ with two molecules of enzyme in the asymmetric unit associated as a ‘pseudo’ dimer (Figure 2A) with a quasi-two fold axis of symmetry; the dimer associates in a parallel ‘*back-to-back*’ arrangement with a buried surface area (BSA) of 3,168 Å^2^ for a total accessible surface area (ASA) of 23,000 Å^2^. The dimer interface involves essentially the hinge regions of the two monomers, the loops between helices α1 and α2 (N-ter domain) and between helix α8 and strand β4 (C-ter domain), the helices α3 and part of the helices α6 of both monomers. The contacts between the monomers consist mainly in polar and charged residues, i.e. Gln80, Asp235, Asp242, Arg71, Arg73, Arg79, Arg187, Arg233 and Arg259. Sulfate ions, present in the crystallization liquor, act as counterions stabilizing this association. The hCD38 structure also revealed the occurrence of a homodimer; however, it is arranged in an anti-parallel ‘head-to-tail’ manner [21], [22]. The dimers of the ADP-ribosyl cyclase from *A. californica* [23] with BSA and ASA values of 2,511 Å^2^ and 22,582 Å^2^ or the human CD157/BST-1 [24] with BSA and ASA values of 5,148 Å^2^ and 21,484 Å^2^ are both organized in a parallel ‘head-to-head’ manner. It is not clear whether any of these association states are biologically relevant, moreover no cooperativity between active sites has been found in kinetic studies on either soluble truncated or detergent-solubilized forms of bovine CD38/NAD^+^glycohydrolase [25], [26]. However glycosylations are likely to hinder a ‘face-to-face’ association, consequently the ‘back-to-back’ glycosylated (and thus more realistic) bCD38 dimeric structures described here are more likely to represent the biologically significant assembly.

These observations are worth discussing within the context of cADPR cellular biology. The head-to-head dimer arrangement observed in the *A. californica* cyclase, was extended by homology to the cell surface anchored hCD38 [23]. This model was since extensively used in literature to solve the ‘topological paradox’ of CD38, suggesting that the ‘cavity’ created by two facing active sites might shield or sequester the reaction product cADPR and thus favor the transport of this Ca^2+^-mobilizing messenger across the cell membrane [17]. As mentioned above, this seems now to be excluded by the crystallographic studies on mammalian CD38. Moreover, although a homodimeric form of CD38 could be demonstrated at the surface of murine B cells [27], the divergent structural data of bovine and human CD38 suggest that such a topological arrangement cannot be based on extensive and specific interactions between their ecto-domains. Finally it remains puzzling that only two transmembrane domains (or four as suggested in some model systems [28]), might serve as a channel specific for a molecule as sophisticated as cADPR, when such known transporters are formed by at least six transmembrane domains [29]. Thus, as suggested before [28], it is much more likely that CD38 reaction products are taken up by cells via nucleoside transporters such as connexin (Cx43) hemichannels [30] rather than by dimers/oligomers of CD38.

### The N-linked glycosylation is near the active site

This is the first structure revealing a *N*-glycosylated form of CD38. A single oligomannose-type sugar antenna, GlcNAc_2_-Man_x_, was expected to be present in the structure of recombinant bCD38. In the crystals, a GlcNAc_2_ unit covalently *N*-linked to the side chain of Asn201 was indeed well defined in the experimental electron density maps, shown in the 2m*Fo*-D*Fc* map in Figure S2. This *N*-glycosylation occurs at a highly conserved residue of the CD38 family of enzymes [13] and is fairly close to the entrance of the catalytic site. The orientation of the carbohydrate antenna is fixed by the stacking of its first N-acetyl glucosamine unit (*NAG1*) to the protein through direct or water-mediated hydrogen bonds and hydrophobic interactions surrounding its N-acetyl group (Figure S3). In contrast, the *NAG2* unit does not interact with the protein. The possibility that the rest of the antenna, which is invisible, might be able to shield the outer surface of the enzyme near the solvent-exposed entry of the active site pocket remains an open question.

### The active site

Residues Trp118, Glu138, Asp147, Trp181 and Glu218 were predicted by homology with hCD38 [31]–[33] to be involved in the molecular mechanism of bCD38. They actually outline its catalytic site, and are all clustered in a pocket located at the junction of the N- and C-ter domains of the protein. The active site of bCD38 consists in the largest solvent accessible cavity detected at the surface of the protein. This 12 Å deep funnel-shaped cavity is also 12 Å wide at its entrance. The total volume of this highly hydrated cavity is about 350 Å^3^ (Figure 2B). A cluster of three strictly conserved phenylalanine residues (Phe117, Phe188 and Phe214) acts as a hydrophobic ‘plug’, sealing the bottom of this pocket. A similar arrangement of Phe residues was recently described in the active site of the sirtuin Sir2Tm [34]. Importantly, the bottom of the active site in bCD38 has distinct structural characteristics: in reason of the tight packing of the protein in this region (residues 116–118, 136–138, 185 and 188) it consists in a very rigid and geometrically constrained system. Moreover the relative accessible surface of the residues at bottom the pocket is very low. For example, one can estimate (NACCESS program) that only 8% of the surface of Glu218 side chain is solvent accessible (Figure S4A). Similarly, the face of the pocket occupied by the conserved residue Trp181 also constitutes a rigid part of the active site.

The invariant Glu218 which was identified as the catalytic residue of bCD38 by site-directed mutagenesis followed by kinetic analysis of the mutants (Table 2 and I. Kuhn et al., to be published) and by use of mechanism-based inhibitors (see below), is located at the very bottom of the active site pocket. This residue is separated from the highly conserved residue Glu138 (which is part of the TLEDTL ‘signature motif’ of the CD38/ADP-ribosyl cyclase enzyme family) by 7.8 Å. This distance can be compared to that of ∼5.5 Å which typically separates the catalytic nucleophile and acid/base pairs of carboxylic acids in the retaining β-glycosidases [35]. The subsite in the vicinity of Glu218 is surrounded by side chains of hydrophobic residues including the Phe ‘plug’ and residues Trp118, Leu137, Ala189, Ile213, Ala219 and Val 217 which are all within a 6 Å radius around its Cε atom. Ser185, whose hydroxyl group is already involved in a hydrogen bond with Trp181 backbone (d = 2.7 Å), is the only other polar residue present in this pocket. It is in proximity with Glu218 at a distance (Oε2^…^O = 3.7–3.8 Å) allowing a low energy hydrogen bond. Interestingly, nonbonded anion-π pairs have recently been suggested to occur in the stabilization of protein structures and in protein-ligand interactions [36]. The edgewise interactions observed between the cluster of buried phenylalanines and the Glu218 side chain (Figure 2B) fulfill the criteria of a stabilizing energy for this carboxylate group. In the unliganded state, Glu218 is also stabilized by water molecules, e.g. two molecules bridge via hydrogen bonds its Oε1 and Oε2 atoms with the backbone amide group of Trp118. Similarly, in the E218Q mutant, both Oε1 and Nε2 atoms of the amide group of Gln218 are interacting with water molecules and the Nε2 is at a distance of 2.9–3.0 Å from the oxygen atom of Ser185 side chain. Several other water molecules are present in this region of the active site of *apo* wt bCD38; four of them are in line and hydrogen bonded to the backbone of the first β-strand of bCD38 C-ter domain between residues Val116 and Lys120. This cluster of water molecules could provide a means of access for bulk water to the bottom of the active site. Among these residues, Trp118 and Lys120 are important in catalysis and binding (see below).

**Table 2:** Site-directed mutagenesis analysis of the role of selected active site residues on the steady-state kinetic parameters of NAD^+^ transformation catalyzed by bCD38.

Enzyme	*K*_m_ (µM)	*k*_cat_ (s^−1^)	relative *k*_cat_	cyclization (%)^a^	*k*_cat_/*K*_m_ (µM^−1^×s^−1^)	relative *k*_cat_/*K*_m_
wild-type	17.1±0.3	57.9±1.9	1	1.1±0.3^b^	3.38±0.13	1
K120A	27.7±3.4	47.1±2.1	0.81	<2	1.70±0.22	0.50
W168A	24.6±4.2	19.6±0.9	0.34	<2	0.8±0.14	0.24
S185A	18.7±2.8	38.2±1.3	0.66	<2	2.04±0.31	0.60
R216A	22.9±3.4	25.4±1.1	0.43	<2	1.1±0.17	0.32
E218Q	24.7±5.7	(3.0±0.2)×10^−2^	0.00052	1.1±0.1^b^	(1.21±0.3)×10^−3^	0.00036

a Conversion of NAD^+^ into cADPR, in percent of the reaction products,

b cADPR was determined using the cycling assay, the other data were obtained using a radiometric HPLC assay with ^14^C-NAD^+^ as substrate.

In *apo* bCD38, the residues of the ‘signature motif’, despite being solvent-exposed, have also a limited conformational flexibility. This is mainly due to the occurrence of an extensive hydrogen bond system that also involves water molecules (Figure S4B). Thus, Glu138 is taking part to a hydrogen-bond network formed with two other highly conserved residues. The Oε1 atom of its side chain is hydrogen bonded (2.9 Å) to N1 of the indole ring of Trp118 and to the Nε2 position (2.9 Å) of His126 side chain. The other atoms, i.e. Oε2 of Glu138 and Nε1 of His126, are all engaged in hydrogen bonds with water molecules. The carboxylate of Asp147 is within a distance of Lys122 ε-amino group to give an ion-pair (∼3.3 Å) and is also surrounded by water molecules.

### The use of 2′-fluorinated analogs of NAD^+^ to trap non-covalent and covalent complexes of bCD38

**Figure 3:**
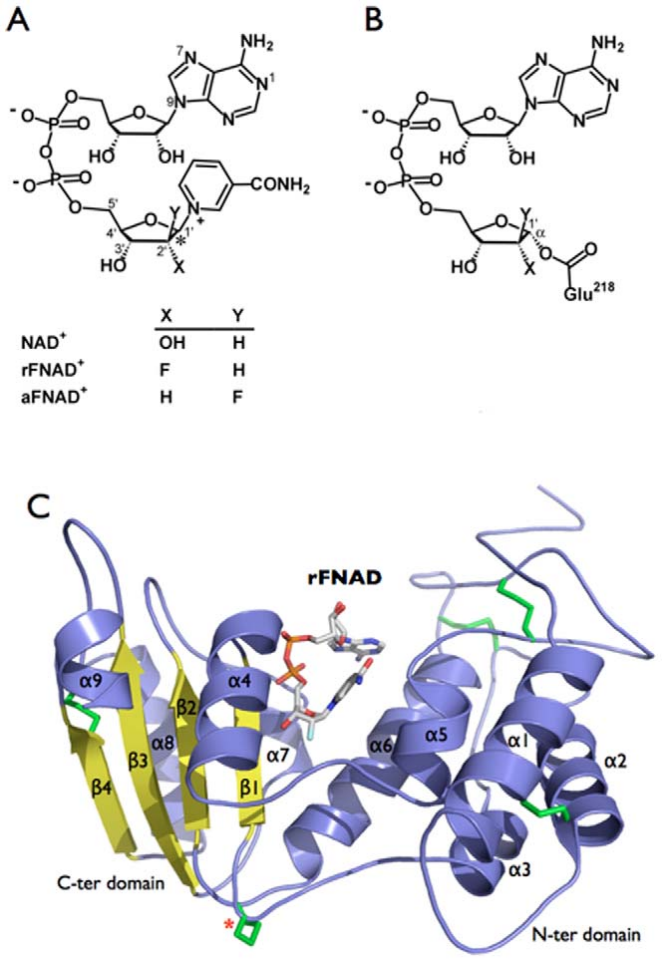
Chemical structure of rFNAD and aFNAD and their corresponding covalent intermediates with Glu218. The 2′-fluorinated analogs of NAD^+^ (A) are slow substrates of bCD38 that can covalently react with the catalytic residue Glu218 (B). The asterisk designates C1′ of the furanoside ring that forms the covalent bond with the carboxylate group of Glu218. The standard numbering is indicated. (C) Overview of the structure of the Michaelis complex with rFNAD. The ligand is shown non-covalently bound in the active site of the bCD38 E218Q mutant.

Soaking crystals of the catalytically impaired E218Q mutant of bCD38 with NAD^+^ failed to capture uncleaved substrate or any of the reaction products, nicotinamide and ADP-ribose. This indicates that the residual activity of bCD38, in which the catalytic Glu218 was mutated to Gln, is still too high (relative *k*_cat_∼1/2000; Table 2) to allow the trapping of the Michaelis complex with the physiological substrate and, importantly, that the active site residues remained catalytically competent in the crystal of the mutant. Next we replaced NAD^+^ with 2′-deoxy-2′-β-D-fluororibofuranoside NAD^+^ (rFNAD), a close (bioisosteric) structural analog carrying a fluorine atom at position 2′ of the ribose moiety linked to nicotinamide (N-ribose) (Figure 3A). As described previously, substitution of the 2′-OH group of N-ribose by a strongly electronegative atom adjacent to the reaction center does not affect the affinity of the analog for the active site of bCD38, but inductively destabilizes the oxocarbenium ion transition state (Figure 1) and impacts the catalytic rate which is considerably reduced (i.e. 4.8×10^5^-fold relative to NAD^+^) [37]. Using this strategy, we were able to trap a non-covalent complex between the E218Q mutant and rFNAD and its structure was solved at 1.94 Å (Table 1). However, for unknown reasons, we were unable to obtain the corresponding Michaelis complex between the mutant and 2′-deoxy-2′-β-D-fluoroarabinofuranoside NAD^+^ (aFNAD). In parallel, we also attempted to trap Michaelis complexes by soaking crystals of wt bCD38 with rFNAD or aFNAD (Figure 3A). However both 2′-fluorinated analogs of NAD^+^ led to high-resolution structures (Table 1) which lacked the nicotinamide ring and where a covalent bond was observed between the carboxylate of Glu218 and C1′ of the 2′-fluorinated N-ribose moieties of the analogs (Figure 3B). The structures of the non-covalent and covalent complexes of bCD38 with rFNAD and aFNAD are described below and the electron density maps of each of the described ligands are shown in Figure S5. They provide invaluable insights into the catalytic mechanism of this enzyme that are also addressed.

At this point it is important to discuss the activity retained by the E218Q mutant of bCD38 (Table 2). It contrasts sharply with the absence of measurable activity exhibited by the homologous E226Q mutant of human CD38 [31] giving access, for this enzyme, to the Michaelis complex with NAD^+^ [38]. We have recently confirmed these data [39] and found that the activity of the E226Q mutant was at least 10^6^-fold inferior to that measured for wt hCD38. However, this residual activity of the E218Q mutant of bCD38 represents a true catalytic activity and not the result of contamination by wild-type enzyme (Materials and Methods) or from the expression system, i.e. no CD38/NAD^+^glycohydrolase-like activity was detectable in supernatants of *Pichia pastoris* cultures. Furthermore it is highly reproducible and a similar range of activity was found with the E218A mutant (I. Kuhn et al., to be published). Finally, in sharp contrast with wt bCD38, the E218 mutants are non-competitively inhibited by pyridine bases (I. Kuhn et al., to be published) ruling out their identity with the wt enzyme. Altogether our results indicate that the E218Q mutant still carries a measurable residual catalytic activity. Moreover the discrepancy between bovine and human enzymes suggests that the reactions catalyzed by these two enzymes might display a different sensitivity towards the mode of action of their catalytic Glu, thus validating the study of bovine CD38.

### Structure of the non-covalent complex of E218Q mutant with rFNAD: the Michaelis complex

Despite the presence of two CD38 molecules in the asymmetric unit of E218Q mutant of bCD38, the structure of the non-covalent complex with rFNAD revealed that only one site is fully occupied; it is possible that one of the monomer and active site are rendered less accessible and/or reactive due to crystalline packing constraints.

Highlighting its conformational rigidity, the active site of bCD38 does not undergo important structural modifications upon rFNAD binding. The backbone position is well conserved; typically, the RMSD computed for the position of Gln218, Glu138, Trp118 and Trp181 backbone atoms is about 0.2 Å when comparing the *apo* E218Q mutant and the Michaelis complex. Only subtle changes could be observed in the side chains positioning. Thus, the most significant one in the buried part of the active site concerns Ile213; two different rotamers are observed in the *apo* enzyme, none being suitable for the placement of the nicotinamide moiety of rFNAD. A third rotamer was indeed selected in the Michaelis complex. The conformation of the Gln218 amide group was also constrained by the bound rFNAD ligand, with adjustments in the torsion angles around Cβ-Cγ (χ_2_) and Cγ-Cδ (χ_3_).

**Figure 4:**
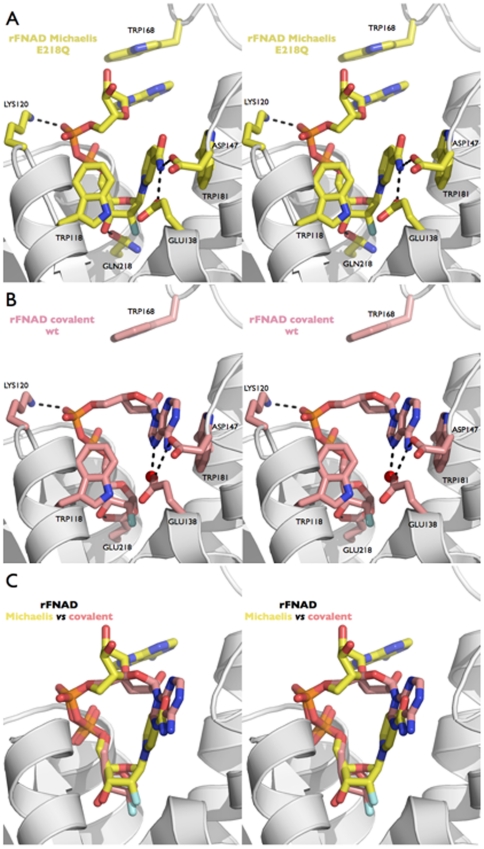
The Michaelis complex and the covalent intermediate trapped with the 2′-ribofluoro analog of NAD^+^. (A) Stereo view showing rFNAD non-covalently bound in the active site of the E218Q bCD38 mutant. Residues critical to substrate binding and catalysis are labeled. Hydrogen bonds are indicated with dashed lines. (B) Stereo view showing the rFNAD substrate covalently bound to residue Glu218 in the active site of the wt bCD38. Residues critical to substrate binding and catalysis are labeled. The water molecule hydrogen bonded to the N7 of the adenine ring and to the side chain of Glu138 is displayed as a red sphere. Hydrogen bonds are indicated with dashed lines. These two views highlight the conformational change undergone by the ligand from the ‘extended’ (A) to the ‘folded’ (B) conformation. The flipping of conserved residue Trp168 accompanies this ligand folding within the active site. (C) Superimposition of the rFNAD ligands observed in the Michaelis (yellow) and covalent (pink) complexes as shown in (A) and (B) respectively. This figure highlights the movement undergone by the adenosine moiety when progressing from the Michaelis complex to the covalent intermediate. The three views are in the same relative orientation.

rFNAD is a bulky ligand (volume ∼176 Å^3^) and its electron density in the non-covalent complex with the E218Q mutant is well defined thus revealing its conformation (Figure 3C). In particular the ribose from the adenosine part is well-resolved in density. This NAD^+^ analog is constrained by the enzyme in a somewhat folded conformation and is stabilized in the active site by extensive interactions (Figure 4A). The distance between C6A and C2N, usually used to define the proximity between the adenine and nicotinamide rings and the folding of NAD(P)^+^ within the active site of enzymes [40], is 9.3 Å in the present Michaelis complex. It thus appears that bCD38 binds rFNAD in a conformation much less extended than the majority of NAD(P)^+^ binding enzymes with Rossmann folds, and other NAD^+^ cleaving enzymes such as diphtheria toxin, sirtuins or ART2.2 where this distance is >12 Å [40].

The active site of bCD38 can be divided into adenosine, pyrophosphate and nicotinamide-ribosyl binding sites. The electron density maps show that the nicotinamide ring of rFNAD is buried in the active site in agreement with the experimental observation that the cleavage of the nicotinamide-ribosyl bond is the first step of catalysis. In the Michaelis complex the leaving group fills a hydrophobic bulge in the upper half of the active site near the signature motif and is positioned through concerted interactions involving strictly conserved residues (Figure 4A). Thus, the N7 atom of its carboxamide group is hydrogen bonded to Oε1 of the carboxylate group of Glu138 (d = 2.7 Å) and Asp147 (at a suboptimal distance = 3.3 Å). This binding pulls the nicotinamide moiety to the bottom of the active site, thus triggering an extensive desolvation of this subsite and the disruption of the hydrogen bond network observed in the unliganded enzyme structures. However, a water molecule remains bridging the O7 of the nicotinamide moiety (d = 2.6 Å) and the amide backbone group of Gly148. Within the complex and in contrast with the *apo* structure, Glu138 is no longer hydrated. Interestingly this residue remains however hydrogen bonded to the indole ring of the conserved residue Trp118 (Oε1^…^N1 d = 2.84 Å); this underlines the rigidity of the active site whose local topology is unaltered by the binding of the nicotinamide-ribosyl moiety of the ligand. The indole ring of Trp118 now stacks with C2′-H and C3′-H on the hydrophobic top-face of the 2′-deoxy-2′-fluororibose ring. Such CH^…^π interactions have been frequently described between tryptophan side chains and donor CH groups polarized by neighboring heteroatoms or halogens [41]. As mentioned above, the Oε2 atom of the carboxylate of Glu138 could be hydrogen-bonded to His126 which thus may serve as a proton relay between the catalytic center and the bulk solvent. As in wt bCD38, the Oδ1 from Asp147 is also bonded to the side chain of Lys122. From a mechanistic perspective, positioning of the nicotinamide moiety by Glu138 and Asp147 in the vicinity of Trp118/Trp181 probably shields the anomeric center of the N-ribose from bulk solvent until breakage of the scissile bond and the exit of nicotinamide out of the active site. Moreover, Trp118 adopts an ideal position to participate to catalysis through cation-π interactions with the oxocarbenium ion that develops in the transition state and in the stabilization of an oxocarbenium ion-like reaction intermediate.

The indole ring of Trp181 is found in proximity of the nicotinamide moiety of bound rFNAD (Figure 4A). This conserved residue was suggested to be involved in hCD38 in the positioning of the nicotinamide moiety of the substrate via π-π interactions [33], [38]. Because of the positively charged pyridinium ring, a more energetic cation-π interaction [42], [43] between the nicotinamide and the indole group should rather be considered. However, from a catalytic perspective such an interaction leading to the stabilization of the ground state (Michaelis complex) would be rather counterproductive. Indeed, stabilization for pyridinium derivatives – resulting in an increase of the leaving group pKa – through cation-π interactions with tryptophan side chain in the ground-state has been well documented [44]. In the rFNAD/E218Q complex, the distance between the pyridinium charge and the centroid of the Trp181 indole ring is equal to 5.25 Å; thus an aromatic cation-π interaction (π^+^-π interaction) between the substrate cationic nicotinamide and this active site residue does not meet the energetically favored parallel-displaced or T-shaped stacking configurations observed between pyridinium derivatives and aromatic rings [43] and is considered unlikely or at best suboptimal. However the topology of the complex illustrated in Figure 4A presents some mechanistic advantages since when the scissile bond becomes elongated in the transition state a parallel-displaced π-π interaction [45], [46] can be expected between the more neutral leaving nicotinamide and the indole ring of Trp181. Thus we suggest that Trp181 instead of stabilizing the substrate in its ground state, which would result in an anticatalytic effect, rather contributes to catalysis by interacting with the leaving group in the transition state. This hypothesis is fully supported by our kinetic studies of mutants affecting Trp181 and Asp147 which show that mutations of these residues affect only *k*_cat_ values but not *K*_m_ (I. Kuhn et al., to be published) which, in bCD38, can be assimilated to the Michaelis complex dissociation constant [25]. Modeling studies indicate that an elongation of the scissile bond by ∼1.0 Å (residual bond order <0.036) increases the interaction between the nicotinamide ring and the pyrrole moiety of the side chain (Supporting Information S1). This movement, which pushes nicotinamide slightly out of the active site, is also accompanied by an optimization of its hydrogen bond with Asp147. A further motion remains necessary to optimize the stacking of nicotinamide onto Trp181 which, by weakening the hydrogen bonds with Glu138 and Asp147, might be instrumental in its departure from the active site.

In the *apo* structure, the catalytic Glu218 forms no salt bridge and, besides the distant Ser185 that it might engage in low energy hydrogen bond, its side chain is only stabilized by water molecules (see above). Upon formation of the Michaelis complex, this hydration network is disrupted and displaced by the binding of the N-ribose moiety of the ligand. As shown in Figure 4A in the rFNAD/E218Q complex the 2′-deoxy-2′-fluororibose linked to nicotinamide is fixed in the active site through efficient hydrogen bonds between its 3′-OH group, which displaces a bound water molecule, and the Oε1 atom of the mutant residue Gln218 (d = 2.7 Å) and backbone NH group of Trp118 (d = 2.9 Å).

The hydrogen-bonding pattern of the fluorine atom in the catalytic site was also investigated. It is commonly accepted that electronegative fluorine is a stronger acceptor than the other halogens but is not as strong as oxygen or nitrogen [47] and C–F bonds are generally poorly solvated. In the complex with rFNAD, the 2′-ribofluoro substituent however interacts with a structural water molecule (d(2′F^…^Wat26) = 2.8 Å) held in position by the carbonyl and amide backbone groups from residues Val116 and Leu137 respectively (Figure S6). This C–F^…^H–O bond, where the fluorine atom is seemingly acting as a hydrogen bond acceptor, is also observed in the covalent complex with rFNAD (see below). Although the existence of such hydrogen bonds in protein/ligand complexes has been controversial [48], their occurrence was recently described with fluorine substituted glycosides where C–F^…^H_2_O interactions were observed on binding to lectins or enzymes [49]. Interestingly, a structural water molecule, with a similar localization in the narrow end of the active site, was also found in hCD38 [38].

The interactions of rFNAD established in the present Michaelis complex can be extrapolated to the binding of NAD^+^ to wt bCD38. As found with similar enzymes such as human CD157 and CD38 [24], [50], the 2′- and 3′-OH groups of the N-ribose of NAD^+^, are expected to make a bidentate interaction with the carboxylate of the catalytic Glu. Moreover, the displacement by these two hydroxyl groups of the ordered water molecules surrounding residue Glu218, might contribute, in a hydrophobic microenvironment, to the binding energy of the substrate by an entropic effect (binding entropy). Comparison of bCD38 E218Q/rFNAD Michaelis complex with that of NMN^+^ bound to the active site of wt hCD38 (PDB code 3dzk; [50]) provided interesting information. Thus, besides some changes in the χ_2_ and χ_3_ torsion angles of Gln218 side chain, which generate a strong hydrogen bond between its Oε atom with the 3′-OH group of the ligand, the absence of a Glu218 and concomitantly the loss of a hydrogen bond with an 2′-OH group, did not significantly affect the positioning of the nicotinamide-ribosyl moiety of the ligand within the active site (Figure S7).

The pyrophosphate backbone of rFNAD, which in general provides great conformational flexibility to NAD^+^, interacts strongly with the protein (Figure 4A). Electrostatic and polar interactions at the entry of the catalytic site involve a basic residue Lys120 (Arg127 in hCD38) and the side chains of the two serine residues Ser119 and Ser212. Of note Lys120 is also a residue which is in contact with *NAG1* of the sugar antenna; however the shortest distance between this proximal sugar and the pyrophosphate bond of the ligand is ∼7.7 Å which is too large for an antenna to affect the access to the active site and ligand binding. Additional interactions with the bCD38 backbone (NH of Ile213 and Phe214) promote anchoring of the substrate in the catalytic cleft. In contrast to 2′-deoxy-2′-fluororibose, the ribose of the adenosine moiety (A-ribose), shows little interactions, if any, with the enzyme and is solvent exposed. This observation is in good agreement with the low discrimination shown by this enzyme between NAD^+^ and NADP^+^ which carries an extra 2′-phosphate group [51]. In one of the two monomers of the asymmetric unit the adenosine moiety appears to be fairly dynamic as demonstrated by the less well-defined electron density and higher thermal factors. The adenine ring is recognized through π-π interactions with the indole ring of the conserved Trp168, a rather flexible residue situated at the outward edge of the active site pocket, and by the guanidinium group of Arg216 interacting with its N7 position. These interactions position the adenine ring in a conformation in which its N1_N_ position is far from the C1′of N-ribose and is thus unsuitable for an efficient ADP-ribosyl cyclase reaction.

### The Michaelis complex and its mechanistic implications

Upon binding the substrate adopts a conformation that buries the nicotinamide-ribose moiety deep into the bottom of the active site and triggers the desolvation of the ribose ring and places its scissile bond within a highly hydrophobic subpocket (C1′ is in the immediate vicinity of Leu137 side chain). The remaining portion of the substrate is positioned towards the entry of the active site cavity which is more accessible to the bulk solvent. In the absence of electrostatic interactions stabilizing the positive charge of the pyridinium ring of the bound substrate, the microenvironment in the immediate vicinity of the scissile bond is characterized by a low dielectric constant. This has several consequences from a mechanistic standpoint. First, as discussed extensively by Buckley et al. [52], the desolvation energy provides a significant driving force for the catalyzed reaction by destabilizing the positively charged nicotinamide ring in the reactant and thus promoting the dissociative cleavage of the nicotinamide-ribosyl bond. Second, as this scissile bond lengthens, the positive charge is gradually transferred from the nicotinamide ring to the N-ribose C1′-O4′ bond generating an oxocarbenium transition state stabilized by electrostatic and cation-π interactions with conserved residues Glu218 and Trp118 and a π-π interaction between the leaving nicotinamide and the indole ring of Trp181. As a result, this ground-state destabilization contributes to catalysis by lowering the energy difference between the reactant and the late transition state characterized by a highly delocalized positive charge and an almost neutral nicotinamide, i.e. in which the nicotinamide-ribosyl bond is almost broken [53]. Finally, the stabilization of the oxocarbenium ion intermediate by Glu218 can occur either by charge-charge stabilization or via a collapse to form a covalent acylal bond at its C1′-position [3]. Importantly, such non-polar microenvironment-related effects could explain the residual catalytic activity of Glu218 mutants of bCD38 such as E218Q (see above). These issues will be fully addressed elsewhere.

In the Michaelis complex Ser185, the only polar residue present in the *apo* wt bCD38 in the vicinity of catalytic Glu218, remains hydrogen bonded to the backbone oxygen of Trp181 (d = 2.56 Å). When compared to the *apo* E218Q mutant, its side chain is now at a larger distance from Nε2 of Gln218 (d = 3.8–4.0 Å) and is also pointing towards the anomeric carbon of the ligand N-ribose (Figure S8). At a distance of 3.4 Å from C1′, the nucleophilic hydroxyl group of Ser185 is somewhat short of a bonding distance which might have been deleterious for catalysis, e.g. resulting in a covalent trapping of the oxocarbenium ion reaction intermediate. But, as suggested before for hCD38 [38], Ser185 might take part to the stabilization of this positively charged transition state/intermediate. However, related to this hypothesis, Ser185Ala mutation of bCD38 did not result in marked changes of its kinetic parameters (Table 2), indicating that the part taken, if any, by the side chain of Ser185 in the stabilization of the transition state of the reaction catalyzed by this enzyme is minimal. This observation contrasts with the results found with hCD38 where mutation of the homologous Ser193 residue resulted in several-fold decreased activities of the mutants [38]. In that enzyme, however, Ser193 was mutated to residues carrying bulkier side chains than the methyl group of Ala; consequently, because they are positioned at a very short distance from the reaction center C1′, they might have disturbed by steric hindrance the precise molecular arrangement of the catalytic groups involved in the cleavage of the scissile bond and thus affected the efficiency of the hCD38 mutants.

### The NMN^+^ moiety of rFNAD adopts a folded and strained conformation in the Michaelis complex - Relevance for catalysis

**Figure 5:**
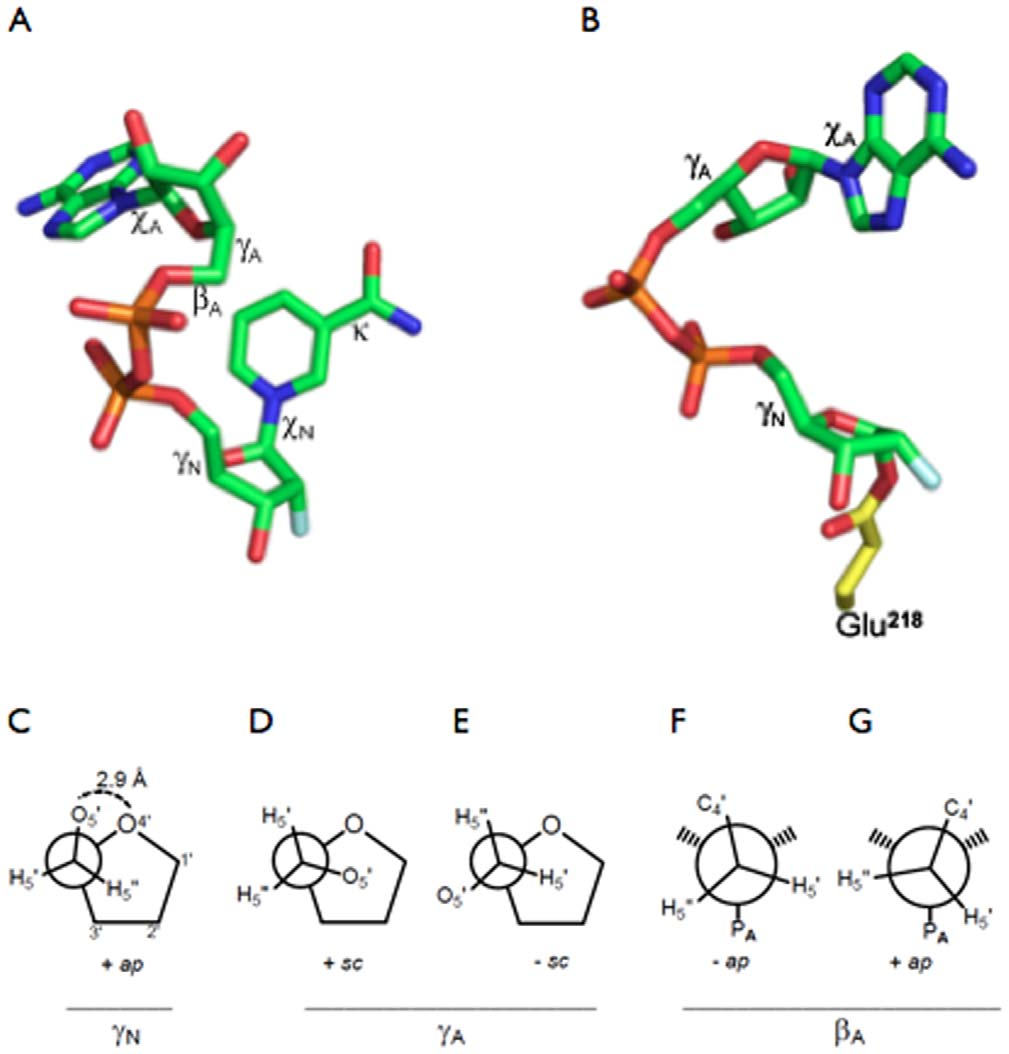
Conformations adopted by rFNAD in the Michaelis and covalent complexes. The conformations of rFNAD as seen in the Michaelis complex trapped in the E218Q mutant (A) and in the corresponding covalent intermediate generated by the wt bCD38 (B). The two views are in the same relative orientation. The different torsion angles are indicated on the structures. Newman projections (C–G) showing conformations of rFNAD in complexes with the active site of bCD38. (C) Rotation around the C4′–C5′ bond of the NMN^+^ moiety of rFNAD in the Michaelis complex (related torsion angle: γ_N_). Comparison of the rotation around the C4′–C5′ bond of the AMP moiety of rFNAD (related torsion angle: γ_A_) in the Michaelis complex (D) and in the covalent complex obtained by reaction with wt bCD38 (E). Comparison of the rotation around the C5′–O5′ bond (related torsion angle: β_A_) of the AMP moiety of rFNAD in the Michaelis complex (F) and in the covalent complex with wt bCD38 (G).

The interactions of the active site residues of bCD38 with rFNAD in the Michaelis complex constrain this slow-substrate in an unusual conformation with regard to its torsion angles. The bound ligand is illustrated in Figure 5A and the values of selected torsion angles that are relevant for the molecular mechanism of bCD38 are given in Table 3. For the nomenclature of the conformers, see IUPAC-IUB recommendations [54].

**Table 3:** Conformational analysis of the ligands bound to the active site of wild-type bCD38 and E218Q mutant.

	NMN^+^ moiety			AMP moiety		
Complex	χ_N_ a (°)	γ_N_ b (°)	κ^c^ (°)	χ_A_ a (°)	γ_A_ b (°)	β_A_ d (°)
E218Q-rFNAD (Michaelis complex)	+145 (*anti*)	+161	−176 (*trans*)	−106 (*anti*)	+54	−168
wt-rFNAD (covalent complex)	NA	+150	NA	NA	NA	NA
		+162		−82	−67	+155
wt-aFNAD (covalent complex)	NA	+158	NA	−84	−75	+175
		+175		−93 (B_e_)^e^	+34	+175
				−73 (B_f_)^e^	−45	+127

a Torsion angle O4′-C1′-N1_N_(or N9_A_)-C2_N_(or C4_A_), defined according to IUPAC-IUB nomenclature.

b Torsion angle O5′-C5′-C4′-C3′.

c Torsion angle C2_N_-C3_N_-C7_N_-O7_N_. The conformation of the carboxamide group is defined as O7_N_ relative to position C2_N_ of the nicotinamide.

d Torsion angle P_A_-O5′-C5′-C4′.

e B_e_ and B_f_ refer, respectively, to the ‘extended’ and ‘folded’ conformations of the covalently linked ligand observed in chain B (see Figure 6).

#### Torsion angles of the nicotinamide 3-carboxamide group and the nicotinamide-ribosyl bond

The hydrogen bond network involving the invariant active site residues Glu138 and Asp147 and the nitrogen of the 3-carboxamide group (Figure 4A) fixes the nicotinamide moiety of the ligand in a conformation where the exocyclic oxygen atom O7 is *trans* relative to the C2 position of the aromatic ring, i.e. torsion angle κ = −176° (Table 3). Moreover, the amide, which makes a low out-of-the plane angle (C4-C3-C7-O7) of ∼4.9°, is approximately coplanar with the pyridine ring. Of note, such a *trans* conformation is prevalent in crystal structures reported for enzyme-bound NAD(P)^+^ cofactors [40]. Related to this finding, *ab initio* calculations have previously indicated that the carboxamide group in free *N*-substituted nicotinamides (such as in NAD^+^) prefers a *cis* orientation whereas in neutral nicotinamide it prefers to be *trans* [55], [56]. This indicates that with respect to the amide group, rFNAD is bound in the Michaelis complex in a high-energy *trans* geometry stabilized by specific interactions. In contrast, the leaving nicotinamide is already in its minimum energy orientation. Consequently, since the cleavage of the scissile bond catalyzed by bCD38 occurs via a very late transition state, i.e. product-like [57], a rate advantage can be expected (Hammond postulate) and estimated to a lowering of the reaction energy by ∼2 kcal.mol^−1^ [56].

The value of the torsion angle about the N-ribosyl bond of rFNAD, χ_N_∼145°, is very unusual compared to the preferred *syn* (60°) or *anti* (−120°) conformations measured in NAD^+^ bound to enzymes as coenzyme [40] or as substrate such as in ADP-ribosyl transferases [58]. This torsion angle is however close to that of NAD^+^ complexed to sirtuins [58]. Such an out of range conformation, which is also dictated by the hydrogen bonds between active site Glu138 and Asp147 and the N7 of the carboxamide group, positions the nicotinamide ring group in an orientation relative to the ribose ring in which steric interactions are expected between the hydrogen atom at C6_N_-position and the ribose oxygen O4′ (d (C6_N_-O4′) = 2.74 Å). Such steric overlaps, which are maximum at χ_N_ = 180°, are known to cause a lengthening of the scissile bond in the ground- state [40], [59] (C1′-N1_N_ = 1.51 Å). Altogether the steric strain in the Michaelis complex caused by the value of χ_N_ can contribute to make nicotinamide a better leaving group, and the ground-state destabilization lower the energy barrier of the reaction.

The occurrence of interactions between the nicotinamide carboxamide moiety with active site acid residues is in excellent agreement with our previous kinetic studies on bovine CD38/NAD^+^glycohydrolase-catalyzed hydrolysis of pyridinium analogs of NAD^+^. We have thus shown that a carboxamide group at position 3 of the pyridine ring provides a hydrolysis efficiency ∼14-fold higher (δΔG^‡^ = −1.65 kcal.mol^−1^ at 37°C) than expected from the pKa of the leaving nicotinamide [53]. Consequently, one could envisage that the combined action of Glu138 and Asp147 residues (Figure 4A) specifically stabilizes a *trans* conformation of the 3-carboxamide substituent and a torsion angle of the nicotinamide-ribosyl bond that increases the reactivity of the scissile bond.

#### Torsion angle about the exocyclic C4′–C5′ bond

Binding of rFNAD to the active site of the enzyme enforces a conformation in which γ_N_, the torsion angle about the C4′–C5′ bond, is ∼161° (Table 3). Rotation about this exocyclic bond plays a crucial role in positioning the 5′-substituent relative to the ribose and nicotinamide rings; interestingly, the +*ap* conformation found for γ_N_ places the exocyclic O5′ within a short contact distance of 2.9 Å above the ribose ring oxygen O4′ (oxygen stack) (Figure 5C). Such a conformation is electrostatically unfavorable in the ground state. Electron donation by a lone pair from the O5′ was suggested to favor the release of electrons from O4′, that accompanies the cleavage of the C1′-N1_N_ bond and the formation of the positively charged oxocarbenium ion [60], [61]. Consequently the proximity between the O5′ and O4′ atoms is expected to enhance the lability of the nicotinamide-ribosyl bond through stabilization of the transition state at the scissile bond cleavage step (see also [62]).

In conclusion, the substrate destabilization occurring in the Michaelis complex, involving steric and electronic constraints (ground-state destabilization), which is relieved en route to the transition state might be part of the arsenal used by bCD38 to cleave the nicotinamide-ribosyl bond.

### Conformation of the AMP moiety of rFNAD adopted in the Michaelis complex

Despite the observed flexibility of this region of the ligand, the adenine ring is observed in an *anti* conformation (χ_A_ = −106°) (Table 3) which corresponds to a conformation strongly preferred by unbound and protein-bound (di)nucleotides [40], [59]. The pucker of the A-ribose (P = 148°) is borderline to the classical C2′-*endo* conformation. Likewise, the torsion angle measured about the exocyclic C4′–C5′ bond, γ_A_ = 54°, corresponds to a staggered +*sc* conformation (Figure 5D) which is predominantly encountered in nucleosides/nucleotides [59]; this contrasts with the −*sc* conformation which seems to be overwhelmingly favored for NAD(P)^+^ bound to the active site of different enzyme families [40]. β_A_ the torsion angle, which defines the rotation about the C5′–O5′ bond, indicated an −*ap* conformation (Figure 5F) which is somewhat less favored than the +*ap* one for enzyme-bound NAD^+^ [40], [59].

Taken together the interactions of the active site residues with the nicotinamide 2′-deoxy-2′-fluororibose moiety of rFNAD constrain this ligand, in the Michaelis complex, in unusual conformations with regard to the N-ribose torsion angles with the leaving pyridine and the C4′–C5′ bond. It seems very likely that the distortions and strain introduced by the binding energy are harnessed by the enzyme to destabilize the scissile bond already in the ground-state and thus contribute to the catalytic mechanism. The AMP moiety displays a much more classical conformation, in line with the rather loose interactions between the active site and this part of the substrate. As such, the adenine ring is in an unfavorable *anti* conformation too far from the reaction center to engage in a cyclization reaction leading to the formation of cADPR.

### Effects of the mutagenesis of active site residues on substrate binding

The structure of the Michaelis complex of bCD38 with rFNAD allows establishing the potential contribution of active site residues to the binding of the substrate. Because the conserved acidic residues Glu138, Asp147 and Glu218 and the hydrophobic residues Trp118 and Trp188 are predominantly involved in the molecular reaction mechanism of the enzyme, their role will be discussed separately (I. Kuhn et al., to be published). Here we will mainly focus on residues Lys120, Trp168 and Arg216 which were found to interact with the AMP moiety of the pseudo-substrate. An alanine scanning mutagenesis was performed and the results are given in Table 2. Surprisingly, none of these residues was found to contribute to the overall binding energy of NAD^+^. Thus the *K*_m_ values are not appreciably affected by the mutation of these residues. Similarly the maximal rates and the percent of NAD^+^ conversion into cADPR did not vary much. Altogether, these results seem to exclude that the interactions found in the crystal between the upper region of the active site pocket and the 5′-AMP moiety of the substrate are individually energetic enough to play a major role in NAD^+^ binding. In fact these data are rather in good agreement with our observations concerning the conformational flexibility of the adenosine moiety of the ligands in the crystals (see below) and also with the poor discrimination shown by bovine CD38/NAD^+^glycohydrolase for NAD^+^ analogs modified in the adenine/adenosine moiety such as NADP^+^, 1,N^6^-etheno NAD^+^ [63] and NGD^+^ [12]. Likewise we have shown previously that although AMP, ADP, ATP and ADP-ribose competitively inhibit this enzyme, the observed inhibition constants were all in the mM range [25], [51]. Altogether these results indicate that the active site of bCD38 is primarily a NMN^+^ binding motif, i.e. the interactions of the active site residues with this positively charged moiety of the substrate are more favorable than those with the AMP moiety, which is largely solvent exposed and conformationally dynamic and which according to our mutagenesis studies makes, surprisingly, only a minor contribution to the overall interaction energy. Thus, in contrast with the NMN^+^ moiety that interacts with a rigid area of the bottom of the active site, the conformation of the AMP part of the ligand observed in the Michaelis complex merely reflects an energy minimum in the crystal rather than energetic interactions with the protein. Moreover, residue Lys120 whose side chain is minimally constrained and solvent exposed can only establish low energy interactions with the pyrophosphate linkage of the ligand.

### Structure of the covalent intermediates obtained by reaction of wild-type bCD38 with rFNAD and aFNAD

The possibility to covalently trap a reaction intermediate by reaction of wt bCD38 with rFNAD and aFNAD ascertains the major role played by the strictly conserved residue Glu218 in the catalytic mechanism of this enzyme. This result also establishes the catalytic competence of the crystallized form of wt bCD38 and the absence of major conformational changes or domain movements during catalysis which might have severely damaged the crystals. It also proves that crystallized wt bCD38 is sufficiently active to cleave the scissile bond of a slow substrate such as rFNAD, which is turned-over by this enzyme about 4.5×10^5^-fold slower than NAD^+^ [37].

Fluorinated glycoside analogs have been widely used as alternative slow substrates and mechanism-based inhibitors providing insights into the structure and molecular mechanism of retaining α- and β-glycosidases, i.e. enzymes known to proceed via a covalent acylal intermediate mechanism [64]. Glycosides substituted at position 2 by a fluorine atom present two major properties: (i) in the absence of a C2-OH group no specific and strong hydrogen bonding can be established with the carboxylate moiety of the enzyme nucleophilic group; the resulting lack of C2-OH polarization which contributes substantially to the stabilization of the oxocarbenium ion-like transition state is much detrimental to the catalytic efficiency of glycosidases [65]; (ii) the presence of the strongly electron-withdrawing fluorine atom adjacent to the reaction center also destabilizes the positively charged oxocarbenium ion transition state by inductive effect. The combination of both effects results in an important decrease of the catalytic rate. One major application of the fluorinated glycosides consists in the trapping, with inversion of configuration, of the covalent reaction intermediate which allows the identification of the nucleophilic carboxylic group, for example by mass spectrometry and X-ray crystallography [64]. Importantly, to be successful this strategy implies that the covalent intermediate is relatively stable and accumulates after the first step (glycosylation) of the catalytic process, i.e. that the deglycosylation step, which leads to the reactivation of the enzyme, is rate-limiting. This proved to be the case for a range of β-glycosidases with substrates having good leaving groups, while remaining more problematic with α-glycosidases because of the relatively fast deglycosylation process [66]. In agreement with observations on hCD38 [67], [68], incubation of crystals of wt bCD38 with rFNAD and aFNAD resulted in stable covalent intermediates whose structures could be resolved at high resolution. This indicates that with rFNAD and aFNAD we were able to manipulate the kinetic parameters of the multistep pathway (Figure 1) and that, in sharp contrast with NAD^+^, the cleavage of their C1′-N1_N_ bonds results in the formation of a covalent intermediate that accumulates because the ensuing deglycosylation step is comparatively much slower, becoming the rate-limiting step of the catalytic process. Importantly, these covalent complexes represent the first image of a reaction intermediate poised for the second step of the catalytic mechanism (solvolysis, cyclization, transglycosidation) (Figure 1) and offer a complete picture of the interactions occurring with the residues of the active site.

In both complexes, residue Glu218 was found covalently linked to the anomeric carbon C1′ of the fluorinated-ribose ring in an axial (α-anomeric) configuration (Figure 4B). This is consistent with a nucleophilic attack of the C1′ atom of the scissile bond of rFNAD and aFNAD by the carboxylate group of the catalytic residue, that occurs with inversion of configuration. The released nicotinamide, as an intermediate product of the reaction, could not be observed in the active site, probably because of its low binding affinity (i.e. mM range [25]) and diffusion away from the catalytic site. In the covalent intermediate, the 3′-OH group is stabilized in both 2′-fluororibose and 2′-fluoroarabinose (chain A) rings through a network of hydrogen bonds with the side chain Oε1 oxygen atom of residue Glu218 (Glu218 Oε1^…^3′O d = 2.7–2.8 Å) and the carbonyl and amide backbone groups from residue Trp118 (Figure 4B). Similar interactions were found between this group and Gln218 in the non-covalent rFNAD/E218Q complex (see above). Subtle differences exist, however, within the covalent complexes obtained with aFNAD. Thus, in contrast to chain A (see above), in the chain B of the dimer, the hydrogen bond between the 3′-OH group of the 2′-deoxy-2′-fluoroarabinoside moiety and Oε1 oxygen atom of Glu218 is no longer observed. Furthermore the carboxylate of Glu218 shifts away from the center of the pocket indicating that the conformation of the side chain of the catalytic residue and that of the 2′fluoroarabinoside are particularly affected in the covalent complex with aFNAD.

Importantly we ascertained that both covalent intermediates were catalytically competent. Although they react extremely slowly with water to produce the hydrolysis products ADP-2′-deoxy-2′-fluororibose and ADP-2′-deoxy-2′-fluoroarabinose, their turnover rate (i.e. reactivation of the enzyme) is significantly accelerated by a suitable alternative acceptor such as methanol which yields the corresponding methanolysis products (C. Cakir-Kiefer, unpublished).

As in the Michaelis complex, the 2′-fluorine atom of rFNAD interacts with a conserved structural water molecule that is hydrogen bonded by the carbonyl and amide nitrogen backbone groups from residues Val116 and Leu137 respectively. In sharp contrast, the 2′F in the aFNAD covalent intermediate is not involved in any bonding. It is too far away from any potential donor or acceptor protein side chain or solvent molecule. The torsion angle γ_N_ determined in the Michaelis complex (+*ap* conformation), was preserved, for both complexes, in the covalently linked 2′F ribosyl rings. Thus, in both cases their O5′ atom remains maintained in a close vicinity with the ring O4′atom.

### Conformational freedom of the AMP moiety of the covalently linked ligands

All crystal structures obtained in this work belong to the orthorhombic space group P2_1_2_1_2_1_ with two molecules in the asymmetric unit (Table 1). Both structures of the covalent complexes revealed differences between chains A or B within the non-crystallographic homodimers. It is not surprising to observe different occupancies or conformations for a same ligand in the active sites of the two distinct enzyme molecules present in the asymmetric unit. Such subtle variations can be explained by different crystalline lattice constraints.

#### The covalent complex with rFNAD

Observation of the two distinct active sites in the dimeric structure of ADP-2′-deoxy-2′-fluororibosylated wt bCD38, reveals an important conformational flexibility of the covalently trapped intermediate. Thus, although both copies of the enzyme contains a covalent adduct formed by reaction between the carboxylate of Glu218 and the C1′ of the N-ribose of rFNAD, the conformation of the covalently bound intermediates are quite different. In one copy of the enzyme (chain B), we were able to model the complete ADP-2′-deoxy-2′-fluororibosyl moiety in a folded conformation with the adenine ring stacked at full occupancy against Trp181. Although the *2Fo-Fc* electron density map is clear in this region, some residual density persists in the *Fo-Fc* electron density map contoured at 2.5σ; this underlines the conformational flexibility in this region of the intermediate. Attempts to refine alternate conformations did not succeed (this is partially caused by a completeness of this specific data set).

In the second copy of the enzyme (chain A), we were only able to model the 2′-deoxy-2′-fluororibose-diphosphate moiety of the covalently bound adduct. Yet the conformation of the intermediate is quite different since we do not see any clear density corresponding to an adenine ring stacked against either residues Trp168 (cf. the conformation in the Michaelis complex) or Trp181 (‘folded conformation’, see below). Both *2Fo-Fc* and *Fo-Fc* maps show residual density (in particular in the nicotinamide binding region) again suggesting the existence of several conformations; however maps are not clear enough to allow unambiguous tracing of the ligands, probably because of rapid interconversion. We therefore did not attempt to refine alternate conformations with different occupancies. Due to the low affinity of nicotinamide towards bCD38 it is unlikely that we could see traces of nicotinamide still trapped in the catalytic pocket; we rather think that we observe an average of conformations that are intermediate between the folded (as seen in the other enzyme copy of the asymmetric unit) and the comparatively more extended conformation revealed by the Michaelis complex.

When the complete ligand could be observed it was found to adopt a ‘folded’ conformation (Figure 4B). In contrast to the more extended conformation observed in the Michaelis complex with rFNAD where the adenine ring stacked against Trp168 (see above), through rotations around its C4′–C5′ and C5′–O5′ bonds the adenosine moiety has flipped over (Figure 5) and the adenine ring is now stacked against residue Trp181, a position close to that previously occupied by the nicotinamide ring in the active site in the non-covalent Michaelis complex (Figure 4A). During this process the orientation around the C4′–C5′ bond (torsion angle γ_A_; Table 3) has changed in both covalent complexes, from *+sc* to *−sc*, corresponding to a rotation of the O5′ and its attached phosphate from above the ribose ring to a distal position (Table 3 and Figure 5E). Similarly β_A_ made a rotation around the C5′–O5′ bond to adopt in both covalent complexes an *+ap* conformation (Table 3 and Figure 5G). In the rFNAD/bCD38 covalent complex the ribose ring of the AMP moiety does not interact with residues of the active site; as in the Michaelis complex, it remains in contact with the solvent and a water molecule (Wat234) interacts with its 2′- and 3′-OH groups. Moreover, its 5′-phosphate remains within bonding distance of the Lys120 side chain. At this point it is of interest to understand the mechanics involved in the movement of the adenine ring. Within the rigid and packed catalytic site, the side chain of Trp181 has no conformational freedom as it is kept in place by water-mediated hydrogen bonds involving the backbone oxygens of Ala 146 and Leu149 and the nitrogen of the indole ring of Trp181. These anchoring interactions are observed in all structures: *apo*, Michaelis complex and covalent complexes regardless of the ligand (rFNAD or aFNAD). Thus, the indole ring of the Trp181 residue is kept in place to act as a rigid platform interacting either with the nicotinamide ring during the scissile bond cleavage step or with the adenine ring in the folded covalent complex. On the other hand, at the solvent exposed entry of the catalytic site, the side chain of Trp168 is fully free to rotate and explore the entire conformational space. This conformational freedom is observed in the crystal structures of the Michaelis and covalent complexes with rFNAD as the side chain of Trp168 adopts two distinct and well-defined conformations. This flipping consists in a 180° rotation around the Ca-Cβ bond that literally accompanies the movement of the adenine from the extended conformation (stacked against Trp168) to the folded conformation (stacked against the rigid Trp181). Furthermore, the substrate being tightly kept in place around its two phosphates, flipping the adenine can thus only be achieved, as observed (Figure 5), through a rotation around the C4′–C5′ and C5′–O5′ bonds of the adenosine moiety. Finally, because Trp168 also blocks the upper part of the active site, such rotation has to be unidirectional.

#### The covalent complex with aFNAD

Comparison of the covalent complexes obtained with rFNAD and aFNAD allows observing subtle differences that distinguish the way the active site of bCD38 accommodates both ligands. With aFNAD, while one active site (chain A) clearly favors a unique conformation similar to the one observed for rFNAD, the second site reveals the presence of two conformations of the covalently trapped intermediate. It is possible that the fluoride atom in aFNAD slows down the ‘adenine flipping’ described above based on the comparison between the Michaelis complex and the covalent-intermediate obtained with rFNAD. This allowed us to capture a snapshot of this fast equilibrium using X-ray crystallography, i.e. to observe the coexistence of both conformers in the same catalytic site. This is a rather rare case since, within the same enzyme molecule, we are able to observe two extreme conformations probably representing two distinct states along the ‘catalytic trajectory’ followed by CD38. The recent structures of hCD38 revealed six slightly different conformers of the same covalent adduct [69] where the substrate already adopts the folded conformation ready for cyclization, which is not the case in our study.

Electron density maps (*2mFo-DFc* and *Fo-DFc*) indicate the presence of two conformations. Attempts to refine simultaneously the two alternate plausible conformations suggest a 1∶1 equilibrium of populations; however, those numbers should be taken with caution, as maps remain noisy even after occupancy refinement of the two alternate conformations (Figure 6). For the indole ring of Trp168, the rather poor electron density map quality clearly indicates a greater conformational freedom for this side chain, as it oscillates between a ‘locked’ conformation (i.e. stacked against the adenine of the ligand as seen in the Michaelis complex) or a more ‘relaxed’ conformation (where the adenine has flipped to stack against Trp181 as seen in the rFNAD covalent complex); this probably results in an averaging and smearing of the electron density in the corresponding area (Movie S1).

**Figure 6:**
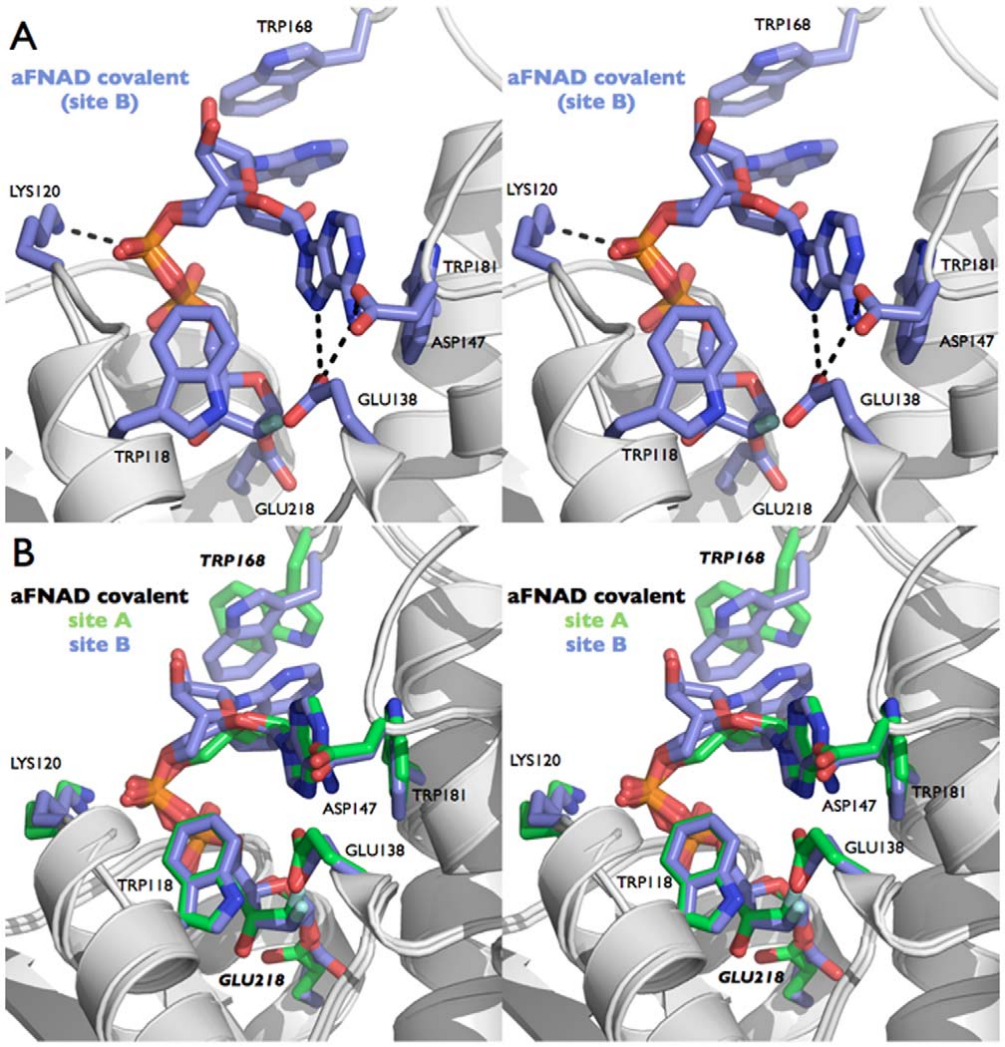
The conformational flexibility of the substrate revealed in the covalent complex between wt bCD38 and aFNAD. (A) Stereo view showing the ‘extended’ and ‘folded’ conformations *simultaneously* observed for the 2′-fluorinated arabinoside covalently bound to the catalytic residue Glu218. Two alternate conformations of the covalent intermediate with aFNAD were trapped within the same catalytic site of wt bCD38 (monomer B). The figure highlights the change of stacking interactions established by the adenine ring after its rearrangement from the more extended conformation (stacked against Trp168) as observed in the Michaelis complex with rFNAD (Figure 4A) to a constrained conformation (stacked against Trp181) as observed in the covalent complex with rFNAD (Figure 4B). Hydrogen bonds are indicated with black dashed lines. (B) Superimposition of the three alternate conformations adopted by aFNAD, one in monomer A (green – stacked against Trp181) and two in monomer B (blue), highlighting the alternate conformations adopted by conserved residues Trp168 and Glu218 (bold labels). Within the two active sites present in the crystallographic asymmetric unit, the two observed ‘extended’ aFNAD conformations are virtually identical. Residues critical to substrate binding and catalysis are labeled. Both views are shown under the same relative orientation.

### Conformational freedom of the AMP moiety and ADP-ribosyl cyclase activity

**Figure 7:**
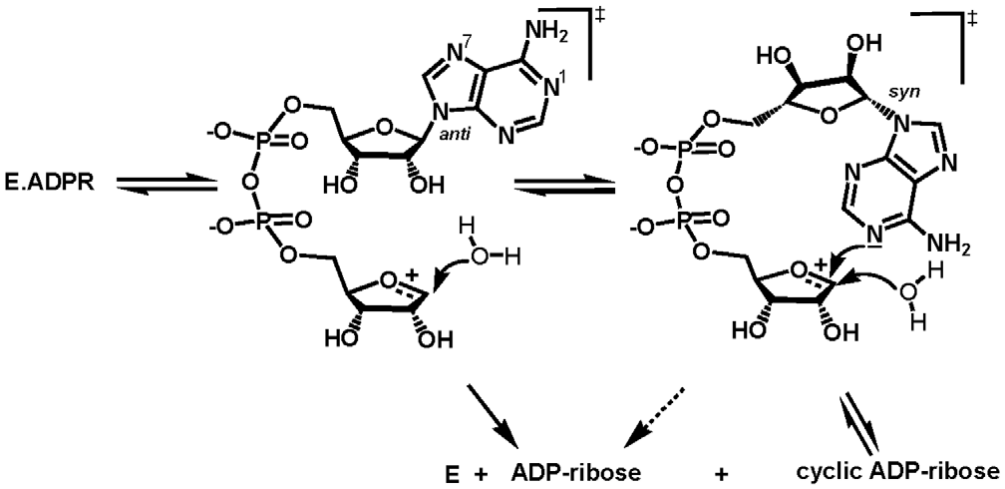
Competition between the NAD^+^glycohydrolase and ADP-ribosyl cyclase activities of bCD38. The formation of cyclic ADP-ribose entails a *syn* conformation of the adenosine moiety in the reaction intermediate bringing the N1 of the adenine ring in the vicinity of the C1′ position of the ribooxocarbenium ion. The thermodynamic equilibrium is in favour of the *anti* conformation.

In all these covalent structures the adenosine ring retains the original thermodynamically favorable *anti*-conformation (see χ_A_ values in Table 3). Consequently, the distance between its N1 position and the anomeric carbon C1′ (about 9.5 Å in the covalent complex with rFNAD) remains too large to make a cADPR derivative. Thus this covalent complex, in which the adenine ring is stacked against Trp181 in an *anti* conformation, is a non-productive one with regard to the ADP-ribosyl cyclase activity of bCD38 (Figure 1). In contrast, a rotation around the N9-C1′ bond of the adenosine moiety, which can take place without collision with the protein, would result in an energetically unfavorable *syn* conformation (χ_A_ changes from −82° to an estimated +77°) and bring the N1 nitrogen at about 3.2 Å of the covalenty bond N-ribosyl C1′. This distance is much more favorable to the cyclization since the positioning of the anomeric carbon of the ribooxocarbenium ion reaction intermediate (Figure 7) is expected to be even closer by 1.5 Å, i.e. at a binding distance of N1 (see below the discussion on the ‘electrophilic migration’ process). However, because the proportion of cADPR formed by CD38 is very low (<2% reaction products), the *syn* conformation is most probably a very minor one and this low population could explain why it is not seen in the crystals. Interestingly, the high yield of cyclization of the surrogate substrate NGD^+^ [70] into cyclic GDP-ribose catalyzed by bCD38 [12] might imply that in the intermediary complex, the guanine ring is also stacked against Trp181 in its energetically favorable *anti* conformation. In that case however, the *anti* conformation is highly favorable because the cyclization process involves the more nucleophilic N7 position of the purine ring [70].

In the covalent complex with rFNAD the adenine ring which is now face-to-face π- stacked against Trp181, is also stabilized within the active site by polar interactions with the side chains of Glu138 and Asp147 which, in the Michaelis complex, were originally hydrogen bonded the nicotinamide amide nitrogen of the substrate (Figure 4B). Thus, the exocyclic N6 amino group gives hydrogen bonds with the Oε1 of Glu138 (d = 2.6 Å) and the Oδ1 of Asp147 (d = 2.8 Å). As mentioned previously, the Oε2 of Glu138 remains hydrogen-bonded to the Nε1 of the imidazole ring of the conserved residue His126. In addition, the N1 atom of the adenine is also hydrogen bonded to the backbone amine of Gly148. Importantly, and in sharp contrast with the Michaelis complex, the entrance of a water molecule within this non-polar environment accompanies the departure of nicotinamide from the active site at the issue of the first step of the reaction process. This molecule (Wat131 in the rFNAD/bCD38 complex) which is ideally poised to play the role of the nucleophilic acceptor in the last – and macroscopically irreversible – step of the catalytic cycle (Figures 1 and 7), is maintained on the top-face of the ribose at about 3.9 Å from the anomeric carbon C1′ by short hydrogen bonds (d = 2.6 Å) with the Oε1 of Glu138 and the N7 of the adenine ring. This contrasts with the adenosine in its *syn* conformation, which establishes less interactions with the active site (N6 hydrogen bonded to the side chain of Glu138) and which fills the space occupied by the putative nucleophilic water molecule. Altogether, it is likely that these interactions also contribute to the stabilization of the *anti* conformation of the adenosine moiety within the active site and consequently may impede the ADP-ribosyl cyclase activity of bCD38. Although CD38 is the only characterized mammalian enzyme able to transform NAD^+^ into the calcium-mobilizing metabolite, cADPR, our structural data strongly suggest that its active site has not been optimized to carry out such a cyclization reaction.

The conformational flexibility of the AMP moiety in the covalent complex uncovered by our structural studies most probably implies that, like the interaction/desorption of the leaving group nicotinamide, the stacking of the adenine ring against Trp181 exists in a rapid on and off equilibrium in this reaction intermediate. This unstacking is required for the adenosine moiety to adopt a *syn* conformation favorable to the cyclization reaction and also for acceptor pyridines to access the anomeric carbon from the top-face of the ribooxocarbenium ion intermediate in order to engage in base-exchange reactions with retention of configuration. It should be noted however that the absence of interactions between residues of the active site of bCD38 with the A-ribose moiety does not allow the enzyme to enforce, via pseudo-rotation, a C2′-*endo* conformation of the ribose which is more favorable to the *syn* conformation of the adenine ring i.e. favorable to cyclization. This might also contribute to the low yield of cADPR.

From a mechanistic perspective, it seems that the active site of CD38 has evolved to initially recognize and bind the NMN^+^ moiety of the NAD^+^ substrate and, in the first step of the catalytic pathway, to catalyze the cleavage of the nicotinamide-ribosyl bond; the motion of the adenosine moiety ring occurs subsequently in a second step. The movement and the stacking of the adenine ring against Trp181 could help to expel nicotinamide from the catalytic site, thus completing the first step of the reaction process and making it nearly irreversible. The conformational change of the ADP-ribosyl intermediate (isomerization step) after cleavage of the scissile bond might also be part of the rate limiting step in the CD38/NAD^+^glycohydrolase catalyzed hydrolysis of NAD^+^. This could explain the results obtained: i) by the group of E. Cordes which has shown that in NAD^+^glycohydrolase-catalyzed hydrolytic cleavage of NMN^+^ and NAD^+^, an α-secondary ^2^H kinetic isotope effect was only observed with the mononucleotide substrate [71], and ii) in the studies on intrinsic fluorescence quenching of native CD38 by (pseudo)substrates [72].

In conclusion, the cyclase activity of bCD38, which involves a rearrangement of the ligand within the catalytic site, represents a remarkable case of steric exclusion between the substrate NAD^+^ and nicotinamide, one of the reaction products, that has to diffuse out of the binding pocket to allow the reaction to proceed. Moreover, within the active site, the adenosine moiety of the substrate must be flexible enough to be able to adopt both *anti* (the great majority seen in the crystal but not competent for the ADP-ribosyl cyclase activity of bCD38) and *syn* conformations. Only an adenosine moiety adopting the energetically unfavorable *syn* conformation would allow the adenine ring stacked against Trp181, to be positioned into a ‘near-attack conformation’ [73] resulting in the formation of cADPR. However, our structural studies show that the *anti* conformation, which is favored thermodynamically [59], [74], is also stabilized by specific interactions with the active site of bCD38. It thus seems that the design of the active site of CD38 is not optimized to make cADPR and that formation of this important metabolite rather results from a stochastic conformational choreography.

### Conclusion

This study represents a comprehensive structural and mechanistic analysis of bovine CD38/NAD^+^glycohydrolase which was successfully crystallized in its *N*-glycosylated form. Compared to the other members of this enzyme family, bCD38 is a shorter polypeptide and lacks an otherwise highly conserved disulfide bond. Despite these differences, the overall three dimensional structure of bCD38 is identical to that of *Aplysia californica* ADP-ribosyl cyclase (1.9 Å Cα rmsd for 30% sequence identity) [23] and hCD38 (1.2 Å Cα rmsd for 48% sequence identity) [21]. In analogy with this latter enzyme, the back-to-back homodimeric bCD38 observed within the asymmetric crystallographic unit also contains a disulfide bond seemingly specific to the mammalian CD38 and stabilizing their hinge region. The active site of bCD38 consists in a funnel-shaped cavity with a fairly rigid structure; it buries the NMN^+^ moiety of the substrate and its scissile bond in its deepest non-polar and least solvent accessible region, whereas the AMP moiety interacts with its more solvent-exposed region, at the entrance of the catalytic cleft. Comparison of the structures of *apo* bCD38 and its non-covalent (Michaelis complex) and covalent complexes obtained in presence the 2′-fluorinated analogs of NAD^+^, rFNAD and aFNAD, provides invaluable insights into the multistep pathway of this enzyme allowing for the dissection of its catalytic mechanism. In the Michaelis complex the binding energy is seemingly used to enforce an unusual and constrained conformation of the NMN^+^ moiety as manifested by (i) the spatial vicinity of the 5′-oxygen with the ribose ring O4′, and (ii) an orientation of the nicotinamide ring that corresponds to a high energy conformation. This ground-state destabilization, in addition to the hydrophobic environment and the desolvation of the nicotinamide-ribosyl bond, promotes the lengthening and cleavage of the scissile bond via a dissociative transition state and the formation of a ribooxocarbenium ion reaction intermediate (Figure 1). This intermediate is electrostatically stabilized by the side chain of Glu218, the catalytic residue, which also plays a paramount importance in catalysis by polarizing the 2′-OH of the substrate NAD^+^. The structures of the covalent intermediates obtained by reaction of wt bCD38 with the slow substrates rFNAD and aFNAD, underline the dynamics of the AMP moiety of the substrates characterized by the movement of the adenine ring which stacks alternatively with Trp168 and Trp181. In all conformations found the adenine ring remains in the thermodynamically most stable *anti* conformation, which is not spatially favorable for the formation of cADPR. The low proportion of the cyclic metabolite formed by this enzyme seems linked to the *anti-syn* equilibrium of the adenosine moiety in the reaction intermediate. It thus appears that the active site of CD38 has not evolved to optimize its ADP-ribosyl cyclase activity relative to the irreversible hydrolytic step leading to ADP-ribose (Figures 1 and 7). Thus, from a purely structural and mechanistic perspective the ADP-ribosyl cyclase activity of CD38 rather seems to correspond to a competitive and opportunistic alternative reaction pathway resulting from a stochastic event. In line with the ‘topological paradox’ of CD38, such a conclusion might seem an additional paradox for the biosynthesis of cADPR, an important secondary messenger in the mobilization of cellular Ca^2+^. Finally, from a structural viewpoint, knowledge of the precise nature of the interactions of bCD38 with its substrates obtained in this study should facilitate structure-based design of specific and efficient inhibitors for this class of enzymes and also provide models for virtual screening.

## Materials and Methods

### Expression and purification of bovine CD38/NAD^+^glycohydrolase

The DNA fragment encoding the ecto-domain (residues 32–278) of bCD38 [13] was cloned into the expression plasmid pPICZαA (Invitrogen) in frame with the yeast α-factor secretion signal sequence under the transcriptional control of the AOX1 promoter and keeping its original stop codon [20]. The recombinant glycosylated enzyme was expressed as a secreted protein in methanol-induced *Pichia pastoris* strains GS115 or X33 [20]. Mutagenesis of bCD38 was done by PCR using the commercial QuikChange Site-Directed Mutagenesis kit (Stratagene). To avoid translational misincorporation during protein synthesis, mutations were systematically obtained by a double-base pair substitution. Soluble wt and mutant secreted bCD38 proteins were recovered from the supernatant of the yeast cultures and purified to homogeneity (SDS-PAGE) by affinity chromatography on a Blue Sepharose 6 Fast Flow CL-6B column (Amersham Biosciences) [20]. To prevent any contamination by wild-type enzyme, each mutant was purified on a dedicated affinity column. Protein concentrations were determined by the OD at 280 nm and by the BCA protein assay (Pierce).

### Crystallization

Bovine CD38 wt or mutant E218Q, both monoglycosylated at position N201 [20], were dialyzed against 20 mM Hepes (pH = 7.4) and concentrated to 5–10 mg/mL for crystallization trials. For the *apo*-enzyme, high-throughput crystallization screening was performed using the nanoliter robotic workstation Mosquito© (TTP LabTech) using vapor-diffusion crystallization technique in hanging drop setups at room temperature. Thirty initial crystallization conditions were found out of the 864 screened. Best crystals grew in 20–30% PEG 4000, 50–250 mM ammonium sulfate and 100 mM sodium cacodylate, sodium acetate or MES at pH = 6.0–6.5 and were flash-frozen in mother liquor supplemented with 20% ethylene glycol or glycerol. Covalent and non-covalent complexes with aFNAD [75] or rFNAD [37] were obtained through 2 to 12 hours soaks at room temperature with 1 to 3 mM ligand followed with a short 2 min soak with the cryo-protection solution also containing 1 to 3 mM ligand.

### X-ray data collection and structure determination and refinement

Diffraction data were collected at the Advanced Light Source beam line 8.3.1 at the Lawrence Berkeley National Laboratory (Berkeley, California). All crystals of the glycosylated form of bovine CD38 belong to orthorhombic space group P2_1_2_1_2_1_ with two molecules per asymmetric unit and diffracted at resolutions ranging from 1.55 to 1.95 Å for an average solvent content of 47%. Within the asymmetric unit, the two monomers are related by a non-crystallographic quasi two-fold axis of symmetry. Data were reduced and scaled using hkl2000/scalepack [76] or elves/scala [77]. The initial *apo* wt bCD38 structure was solved at 1.8 Å resolution by molecular replacement with Phaser [78] using the hCD38 structure (PDB code 1yh3) as search probe. Following the initial molecular replacement solution, automatic building using arp/warp [79] followed by refinement using refmac [80] then phenix [81] and manual model building in coot [82] yielded the final structure including residues Ser37 to Leu275 and Gly38 to Arg277 for monomers A and B respectively, together with water molecules. To minimize model bias, the mutant and all liganded structures reported in this study were solved by molecular replacement using our *apo* wt bCD38 structure and refined in phenix. The bCD38mt in its *apo* form or bound to aFNAD respectively diffracted to 1.55 Å and 1.58 Å resolution whereas the form bound to rFNAD diffracted to 1.94 Å. The wt bCD38 bound to aFNAD and rFNAD respectively diffracted to 1.95 Å and 1.78 Å resolution. For the mutant E218Q bCD38 enzyme bound to rFNAD, observation of the initial Fourier synthesis *Fo*-*Fc* difference map derived from molecular replacement revealed density corresponding to the non-covalently bound ligand (Michaelis complex). For wt bCD38 bound to aFNAD or rFNAD, observation of the initial Fourier synthesis *Fo*-*Fc* difference map derived from molecular replacement revealed density corresponding to the ligand covalently bound to the strictly conserved residue Glu218 in the catalytic site. In order to investigate the subtle differences between the two catalytic sites, non-crystallographic symmetry averaging was NOT applied during refinement. Ligands were generated using eLBOW and were explicitly included when refinement of the protein model showed reasonable convergence. For wt bCD38 bound to aFNAD, two alternate conformations of aFNAD were modeled (in chain B) and their relative occupancies were refined in PHENIX. In all liganded structures, all sites appear to be fully occupied. The electron density maps of the ligands are shown in Figure S5. Alternate conformations of side-chains were also included. In all structures the two first N-Acetyl glucosamine units of the N-linked carbohydrate chain at residue N201 were observed. The quality of each structure was evaluated using MolProbity [83]. The figures were generated with pymol [84].

### Enzyme assays and kinetic studies

The enzyme activity was measured as described previously [14], [85], [86]. Steady state kinetic studies were performed accordingly. Briefly, the enzyme was incubated at 37°C with NAD^+^ (5–400 µM) and 2.5×10^5^ dpm [*adenosine*-U-^14^C] NAD^+^ (Amersham Biosciences) in 200 µL (final volume) 10 mM potassium phosphate buffer at pH 7.4 (buffer A). At selected times, 50 µL aliquots were removed and treated with ice-cold perchloric acid (2% v/v final concentration) to stop the reaction. After neutralization with K_2_CO_3_, the precipitated proteins were removed by centrifugation and product formation was monitored by HPLC.

Analysis of the reaction products was performed on a 300×3.9-mm µBondapak C_18_ column (Waters Assoc.). The isocratically eluted compounds (10 mM ammonium phosphate buffer at pH 5.5 containing 0.8–1.2% (v/v) acetonitrile; flow rate: 1 mL/min) were detected by absorbance recordings at 260 nm and by radiodetection (Flo-one, Packard Radiometric Instruments) when using [^14^C] NAD^+^. Peaks were identified by comparison with authentic samples, and areas obtained from UV recordings were normalized using the molar extinction coefficients of the reaction products. When indicated cADPR was also determined using a cycling assay [85].

Kinetic parameters K_m_ and k_cat_ were determined from the plots of the initial rates of product(s) (ADP-ribose+cyclic ADP-ribose) formation as a function of substrate concentration (8 data points) according to Michaelis–Menten kinetics, using a GrapPad Prism nonlinear curve fitting program (GrapPad Software). Alternatively NAD^+^glycohydrolase activity was determined by a sensitive continuous fluorometric method using 1,N^6^-etheno NAD^+^ (ε-NAD^+^, Sigma) as substrate, as previously described [4]. This assay was performed in a thermostatically controlled fluorimeter cuvette at 37°C in 1 mL (final volume) of buffer A. It consists of measuring an increase of fluorescence (Shimadzu, RF 5301 PC) at λ_em_ = 410 nm (λ_exc_ = 310 nm) resulting from the hydrolytic conversion of the dinucleotide to 1,*N* ^6^-etheno ADP-ribose.

## Supporting Information

Figure S1 **Structure highlighting the domain lacking in the hydrosoluble Δα1 bovine CD38/NAD^+^glycohydrolase.**
(PDF)

Figure S2 **The** ***N*** **-glycosylation site in bCD38.**
(PDF)

Figure S3 **Detail of the enzyme-carbohydrate interactions.**
(PDF)

Figure S4 **Details of the active site of wild-type** ***apo*** **bCD38.** A - Bottom of the active site in the vicinity of the catalytic residue Glu218. B - The hydrogen bond network in the ‘signature motif’ in the vicinity of Glu138.
(PDF)

Figure S5 **Representative maximum-likelihood weighted 2m** ***Fo-DFc*** **Fourier difference electron density maps contoured at 1σ showing the ligands bound in the active site of bCD38.** (A) non-covalent rFNAD (Michaelis complex) and (B) covalent rFNAD.
(PDF)

Figure S6 **Michaelis complex: interaction of the 2′-F atom of rFNAD with a structural water molecule of bCD38.**
(PDF)

Figure S7 **Positioning of the nicotinamide-ribosyl ring in the active site of human and bovine CD38.**
(PDF)

Figure S8 **Positioning of Ser185 relative to Glu/Gln218 and to ribosyl C1′.**
(PDF)

Movie S1 **Conformational rearrangement undergone by the ligand in the covalent complex obtained with aFNAD.** It involves the repositioning of its adenine ring from a solvent-exposed position stacked against Trp168 (ligand in blue) to a more buried position stacked against Trp181 (ligand in green).
(MOV)

Supporting Information S1 **Nature of the interactions between nicotinamide and Trp181 indole ring in bCD38.**
(PDF)
