# Supplementary material for: Insights into the Mechanism of Bovine CD38/NAD+Glycohydrolase from the X-Ray Structures of Its Michaelis Complex and Covalently-Trapped Intermediates
Source: PLoS One. 2012 Apr 18;7(4):e34918. doi: 10.1371/journal.pone.0034918 (PMC3329556; doi:10.1371/journal.pone.0034918)
Supplement: Figure S1 — Structure highlighting the domain lacking in the hydrosoluble Δα1 bovine CD38/NAD+glycohydrolase. (PDF) [file pone.0034918.s001.pdf]

Supporting Information

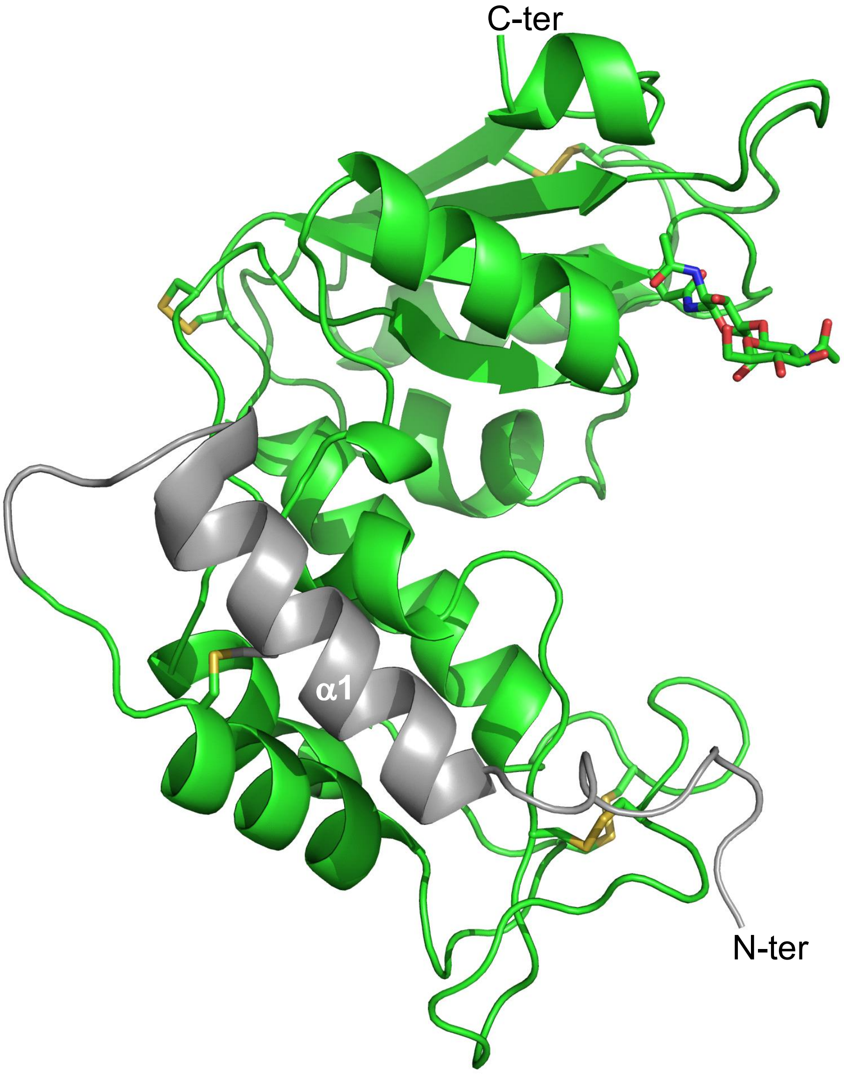

Fig. S1 Structure highlighting the domain lacking in the hydrosoluble Δα1 bovine CD38/NAD$^+$glycohydrolase. Before recombinant forms of bovine ecto-CD38/NAD$^+$glyco-hydrolase became available, this type II plasma membrane-bound protein was purified to homogeneity after solubilization with detergents [1] or by treatment with steapsin, a crude form of pancreatic lipase [2]. We have demonstrated that this latter technique, which was early on widely used to solubilize mammalian NAD$^+$glycohydrolases, see e.g. [2,3], involves a proteolytic cleavage at Arg71 and the loss of a rather large domain of 71 residues at the N-terminal region. These N-ter residues encompass the short intracellular and transmembrane domains (31 residues) and a juxtamembrane domain (40 residues) of the native protein [4]. Accordingly, the hydrosoluble truncated Δ71 bovine CD38/NAD$^+$glycohydrolase, which is fully catalytically active, represents the smallest functional domain (207 amino acids) endowed with all the catalytic activities so far identified. for this family of enzymes.

Moreover the deletion of about 40 residues in the juxtamembrane domain of bCD38 - corresponding to the $\alpha 1$ helix highlighted in grey - does not affect the overall stability of the architecture of this protein nor its active site.

References

1. Muller-Steffner H, Schenherr-Gusse I, Tarnus C, Schuber F (1993) Calf spleen NAD^+glycohydrolase: solubilization, purification, and properties of the intact form of the enzyme. Arch Biochem Biophys 304: 154-162.
2. Schuber F, Travo P (1976) Calf spleen nicotinamide adenine dinucleotide glycohydrolase. Solubilization, purification and properties of the enzyme. Eur J Biochem 65: 247-255.
3. Swislocki NI, Kalish MI, Chasalow FI, Kaplan NO (1967). Solubilization and comparative properties of some mammalian diphosphopyridines nucleosidases. J Biol Chem 242: 1089-1094.
4. Augustin A, Muller-Steffner H, Schuber F (2000) Molecular cloning and functional expression of bovine spleen ecto-NAD^+glycohydrolase: structural identity with human CD38. Biochem J 345: 43-52.
