# Supplementary material for: Insights into the Mechanism of Bovine CD38/NAD+Glycohydrolase from the X-Ray Structures of Its Michaelis Complex and Covalently-Trapped Intermediates
Source: PLoS One. 2012 Apr 18;7(4):e34918. doi: 10.1371/journal.pone.0034918 (PMC3329556; doi:10.1371/journal.pone.0034918)
Supplement: Figure S2 — The N -glycosylation site in bCD38. (PDF) [file pone.0034918.s002.pdf]

Supporting Information

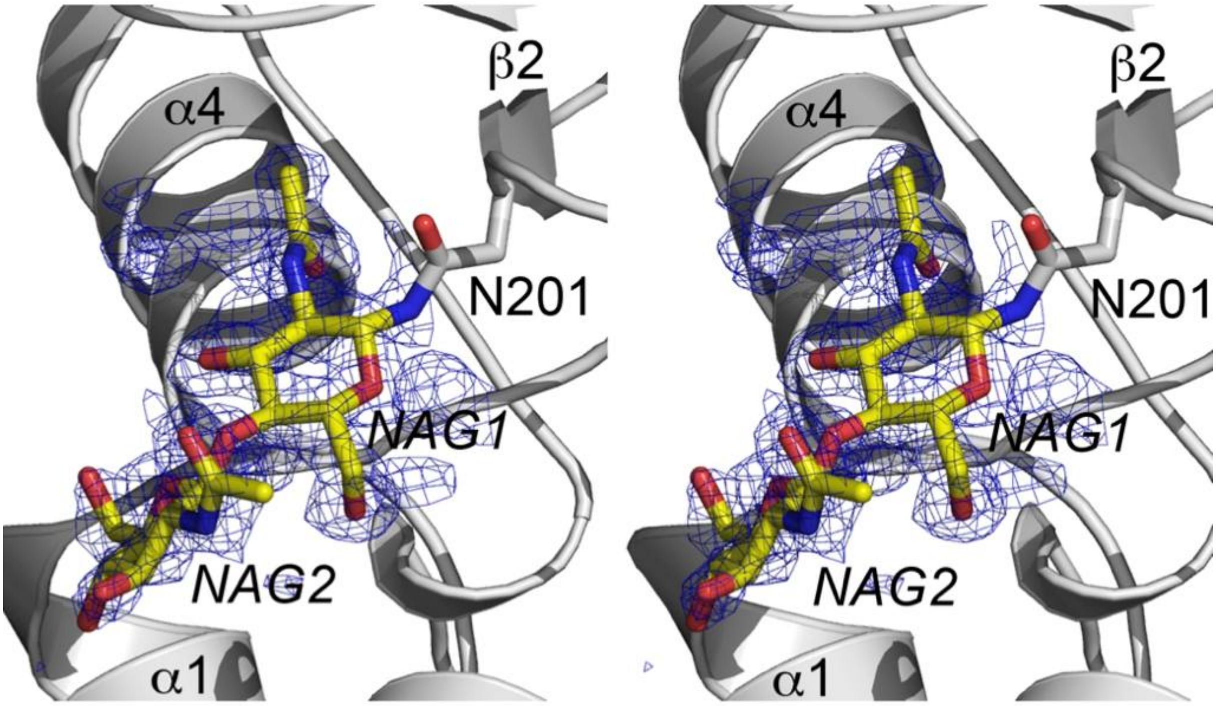

Fig. S2 The *N*-glycosylation site in bCD38. Stereo view showing the maximum likelihood-weighted 2mFo-DFc electron density Fourier difference map contoured at 1.3σ. Two N-acetyl-glucosamine units (*NAG1* and *NAG2*) bound at Asn201, the single glycosylation site present on bCD38, are well resolved in the final model. The side chain of residue Asn201 is shown. For the sake of clarity, the electron density is only displayed around the carbohydrate chain. Secondary structure elements are labeled.
