# Supplementary material for: Insights into the Mechanism of Bovine CD38/NAD+Glycohydrolase from the X-Ray Structures of Its Michaelis Complex and Covalently-Trapped Intermediates
Source: PLoS One. 2012 Apr 18;7(4):e34918. doi: 10.1371/journal.pone.0034918 (PMC3329556; doi:10.1371/journal.pone.0034918)
Supplement: Figure S3 — Detail of the enzyme-carbohydrate interactions. (PDF) [file pone.0034918.s003.pdf]

Supporting Information

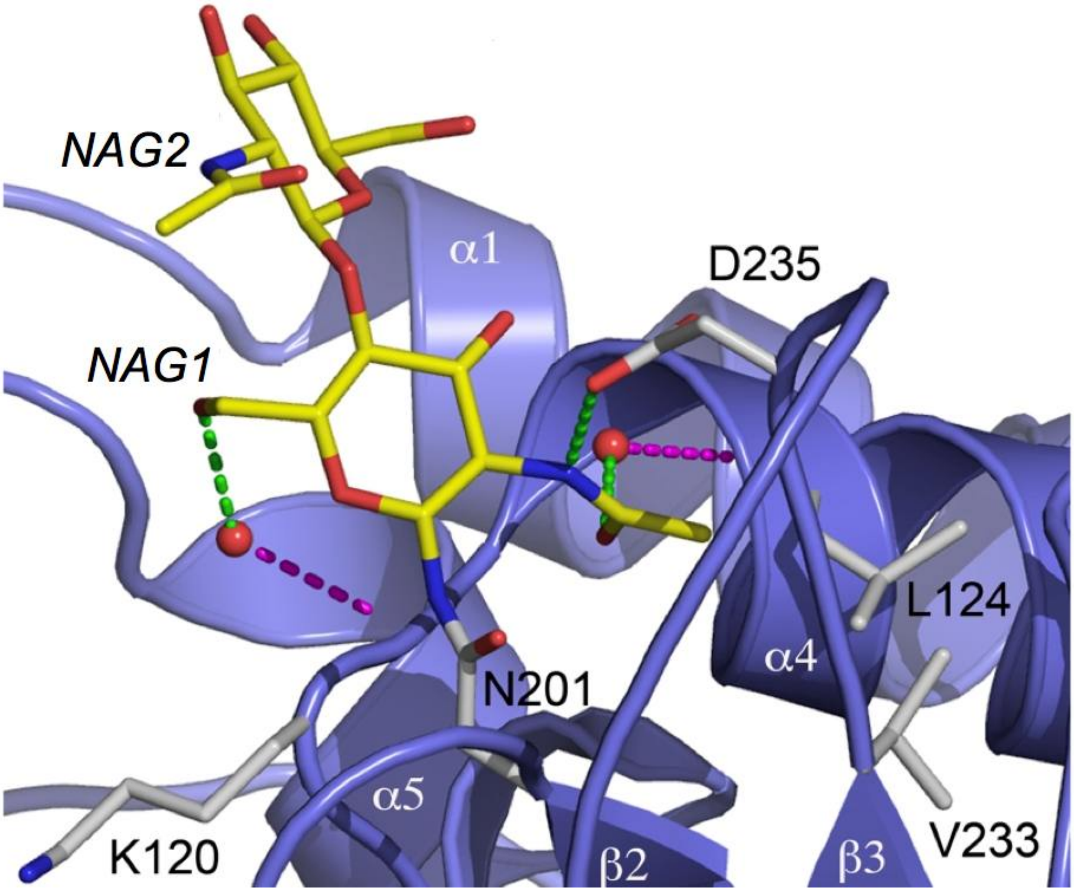

Fig. S3 Detail of the enzyme-carbohydrate interactions. The orientation of the carbohydrate antenna is fixed by the stacking of its first *N*-Acetyl glucosamine unit (*NAG1*) to the protein through direct or water-mediated hydrogen bonds and hydrophobic interactions surrounding its *N*-acetyl group. The nitrogen atom is hydrogen bonded with the Asp235 side chain while its methyl group sits within a hydrophobic cavity contributed by Leu124 and Val233. Two water-mediated interactions between the protein backbone and the *NAG-1* unit also contribute to its stabilization at the surface of the enzyme: a first one between the carbonyl of residue Lys120 and the O6 hydroxyl group and a second one between the amide of residue Leu124 and the CO of the N-acetyl group. Residue Lys120 is positioned within the protein structure by a hydrogen bond between its backbone CO with Nδ of Asn201 and by an ionic interaction between its side chain with the carboxylate of Asp209. In addition a hydrogen bond is linking the 3OH of *NAG1* to the guanidinium group of Arg271, a residue also in ionic interaction with Glu123 and Asp235. In contrast, the *NAG2* unit does not interact with the protein.
