# Supplementary material for: Insights into the Mechanism of Bovine CD38/NAD+Glycohydrolase from the X-Ray Structures of Its Michaelis Complex and Covalently-Trapped Intermediates
Source: PLoS One. 2012 Apr 18;7(4):e34918. doi: 10.1371/journal.pone.0034918 (PMC3329556; doi:10.1371/journal.pone.0034918)
Supplement: Figure S4 — Details of the active site of wild-type apo bCD38. A - Bottom of the active site in the vicinity of the catalytic residue Glu218. B - The hydrogen bond network in the ‘signature motif’ in the vicinity of Glu138. (PDF) [file pone.0034918.s004.pdf]

Supporting Information

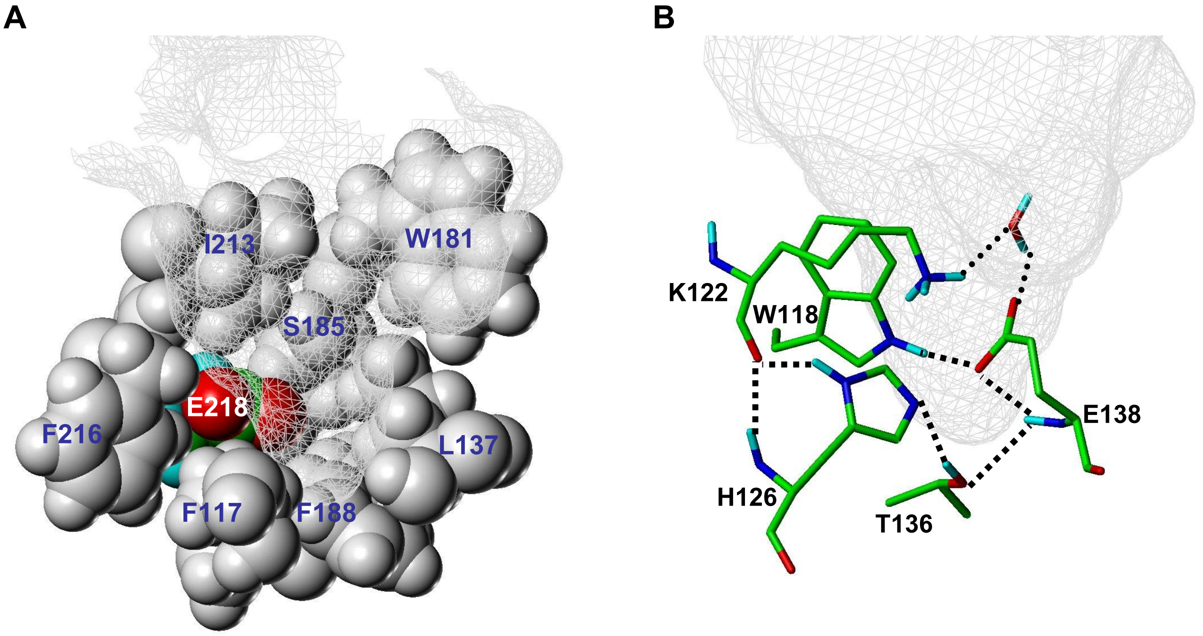

Fig. S4 Details of the active site of wild-type *apo* bCD38

A - Bottom of the active site in the vicinity of the catalytic residue Glu218. The side chains of Glu218 and of surrounding residues, displayed using a space-filled representation, are tightly packed. The oxygen, nitrogen, carbon and hydrogen atoms of Glu218 are colored in red, blue, green and cyan, all atoms of other residues are colored in grey.

B - The hydrogen bond network in the 'signature motif' in the vicinity of Glu138. The solvent-exposed "signature motif" TLEDTL (residues 136-141) is maintained by up to 8 hydrogen bonds between 5 residues. Thus, the carboxyl group of Glu138 is hydrogen bonded to its own backbone amide group, to the amine of Lys122 via a conserved water molecule and to the indole group of Trp118. The backbone amide of Glu138 is also hydrogen bonded to the side chain hydroxyl group of Thr136. The backbone amide and Nδ hydrogens of His126 are hydrogen bonded to the backbone carbonyl group of Lys122. Finally, the Nε hydrogen of His126 is also hydrogen bonded to the hydroxyl group of Thr136. The oxygen, nitrogen, carbon and hydrogen atoms are displayed using red, blue, green and cyan capped sticks, respectively.

A and **B** : The solvent accessible surface (Connolly surface) of the active site is depicted as a gray mesh.
