# Supplementary material for: Insights into the Mechanism of Bovine CD38/NAD+Glycohydrolase from the X-Ray Structures of Its Michaelis Complex and Covalently-Trapped Intermediates
Source: PLoS One. 2012 Apr 18;7(4):e34918. doi: 10.1371/journal.pone.0034918 (PMC3329556; doi:10.1371/journal.pone.0034918)
Supplement: Figure S5 — Representative maximum-likelihood weighted 2m Fo-DFc Fourier difference electron density maps contoured at 1σ showing the ligands bound in the active site of bCD38. (A) non-covalent rFNAD (Michaelis complex) and (B) covalent rFNAD. (PDF) [file pone.0034918.s005.pdf]

Supporting Information

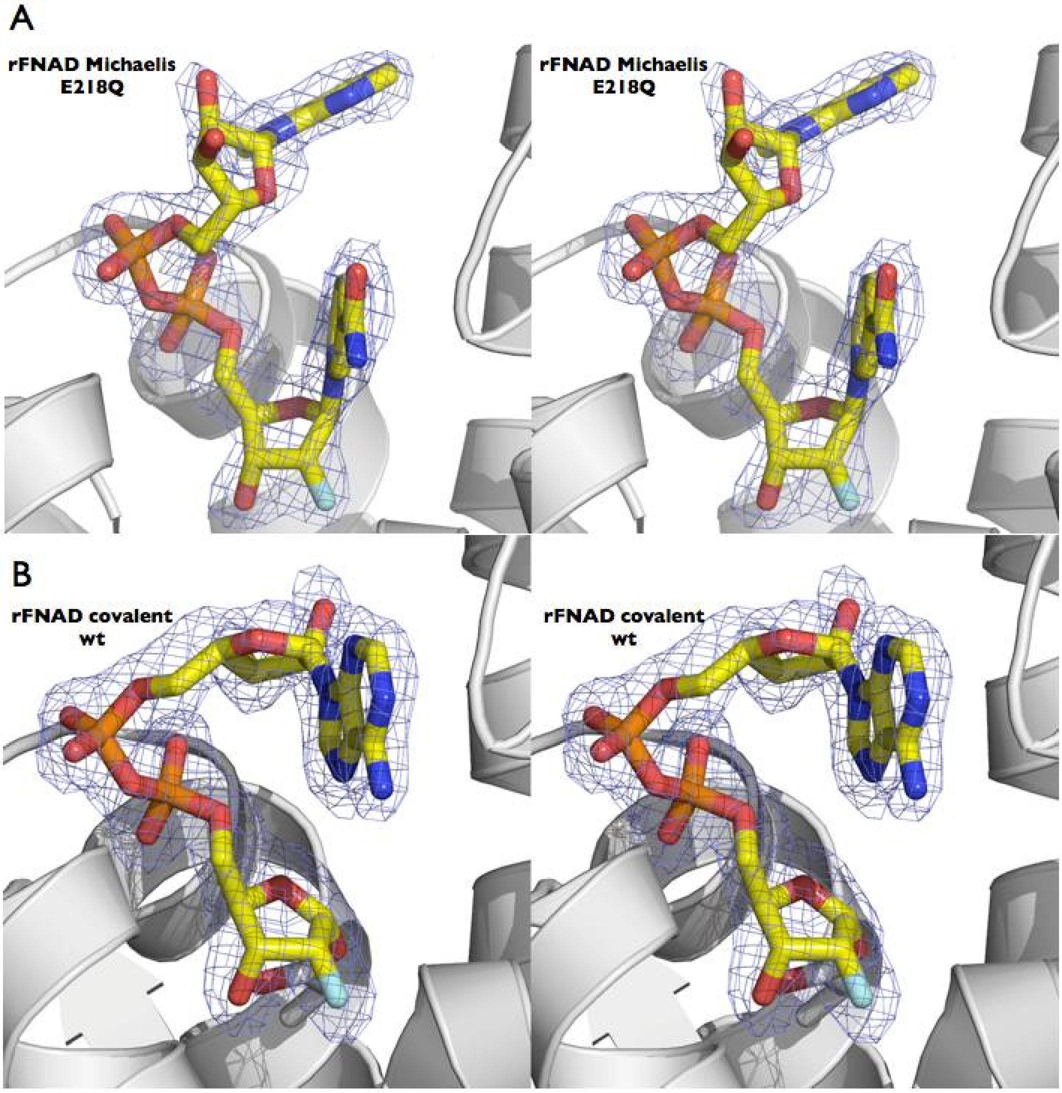

Fig. S5 Representative maximum-likelihood weighted 2mFo-DFc Fourier difference electron density maps contoured at 1σ showing the ligands bound in the active site of bCD38. (**A**) non-covalent rFNAD (Michaelis complex) and (**B**) covalent rFNAD. The orientation is similar to the one in Figures 4 and 6.
