# Supplementary material for: Insights into the Mechanism of Bovine CD38/NAD+Glycohydrolase from the X-Ray Structures of Its Michaelis Complex and Covalently-Trapped Intermediates
Source: PLoS One. 2012 Apr 18;7(4):e34918. doi: 10.1371/journal.pone.0034918 (PMC3329556; doi:10.1371/journal.pone.0034918)
Supplement: Figure S6 — Michaelis complex: interaction of the 2′-F atom of rFNAD with a structural water molecule of bCD38. (PDF) [file pone.0034918.s006.pdf]

Supporting Information

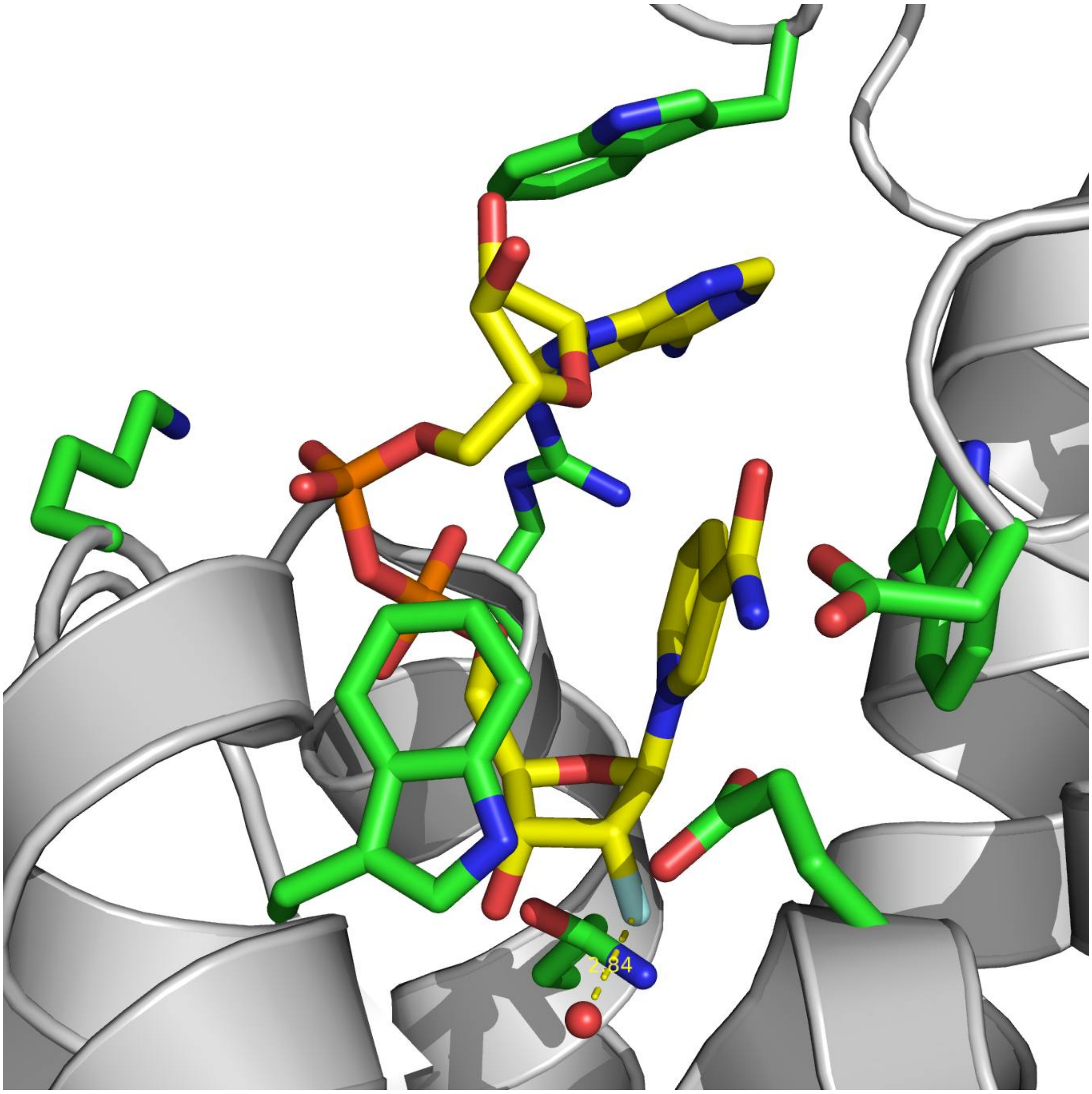

Fig. S6 Michaelis complex : interaction of the 2'-F atom of rFNAD with a structural water molecule of bCD38 (red sphere). The calculated distance is 2.84 Å.
