# Supplementary material for: Insights into the Mechanism of Bovine CD38/NAD+Glycohydrolase from the X-Ray Structures of Its Michaelis Complex and Covalently-Trapped Intermediates
Source: PLoS One. 2012 Apr 18;7(4):e34918. doi: 10.1371/journal.pone.0034918 (PMC3329556; doi:10.1371/journal.pone.0034918)
Supplement: Figure S7 — Positioning of the nicotinamide-ribosyl ring in the active site of human and bovine CD38. (PDF) [file pone.0034918.s007.pdf]

Supporting Information

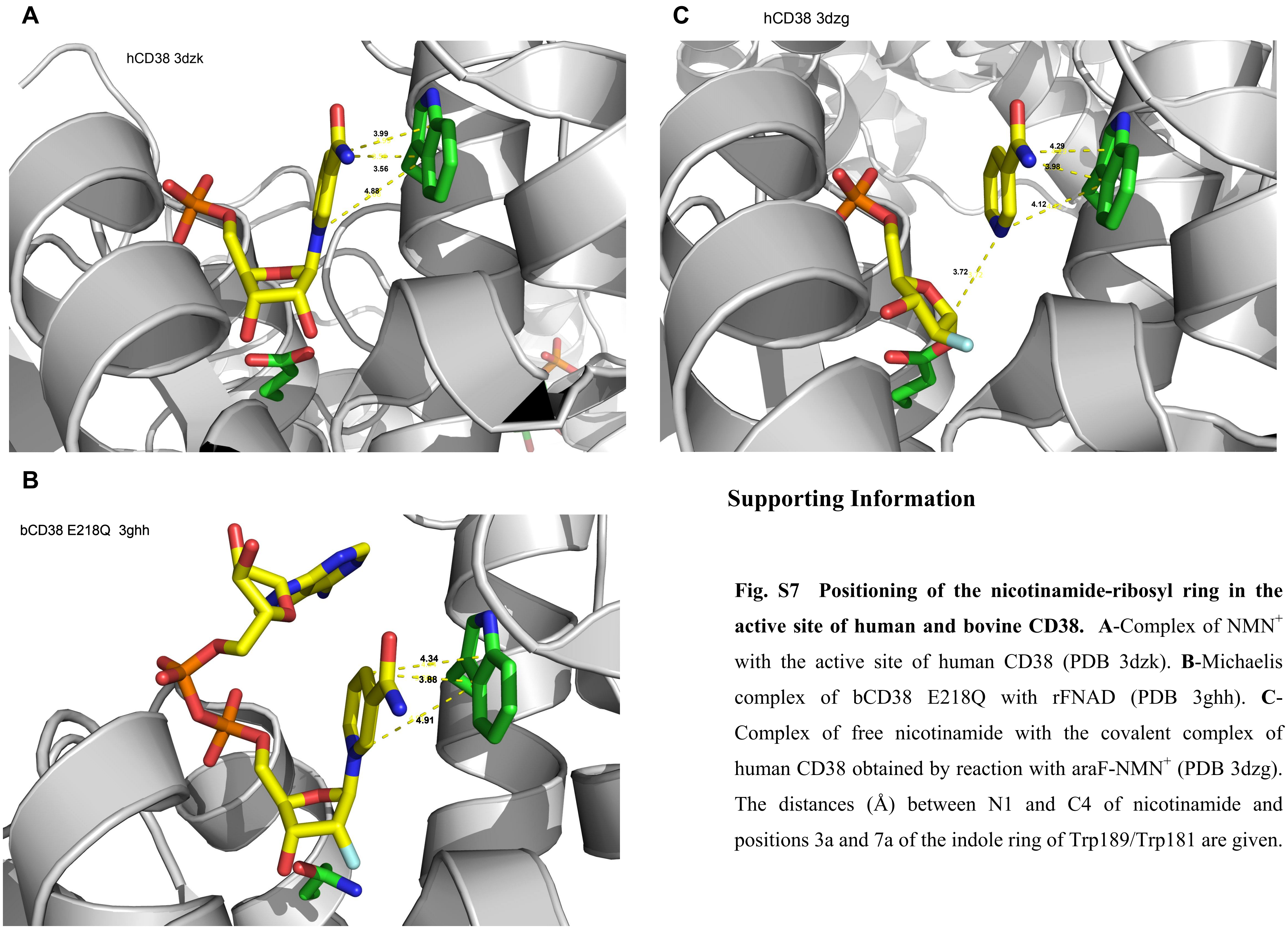

Fig. S7 Positioning of the nicotinamide-ribosyl ring in the active site of human and bovine CD38. **A**-Complex of NMN^+ with the active site of human CD38 (PDB 3dzk). **B**-Michaelis complex of bCD38 E218Q with rFNAD (PDB 3ghh). **C**-Complex of free nicotinamide with the covalent complex of human CD38 obtained by reaction with araF-NMN^+ (PDB 3dzg). The distances (Å) between N1 and C4 of nicotinamide and positions 3a and 7a of the indole ring of Trp189/Trp181 are given.
