# Supplementary material for: Insights into the Mechanism of Bovine CD38/NAD+Glycohydrolase from the X-Ray Structures of Its Michaelis Complex and Covalently-Trapped Intermediates
Source: PLoS One. 2012 Apr 18;7(4):e34918. doi: 10.1371/journal.pone.0034918 (PMC3329556; doi:10.1371/journal.pone.0034918)
Supplement: Figure S8 — Positioning of Ser185 relative to Glu/Gln218 and to ribosyl C1′. (PDF) [file pone.0034918.s008.pdf]

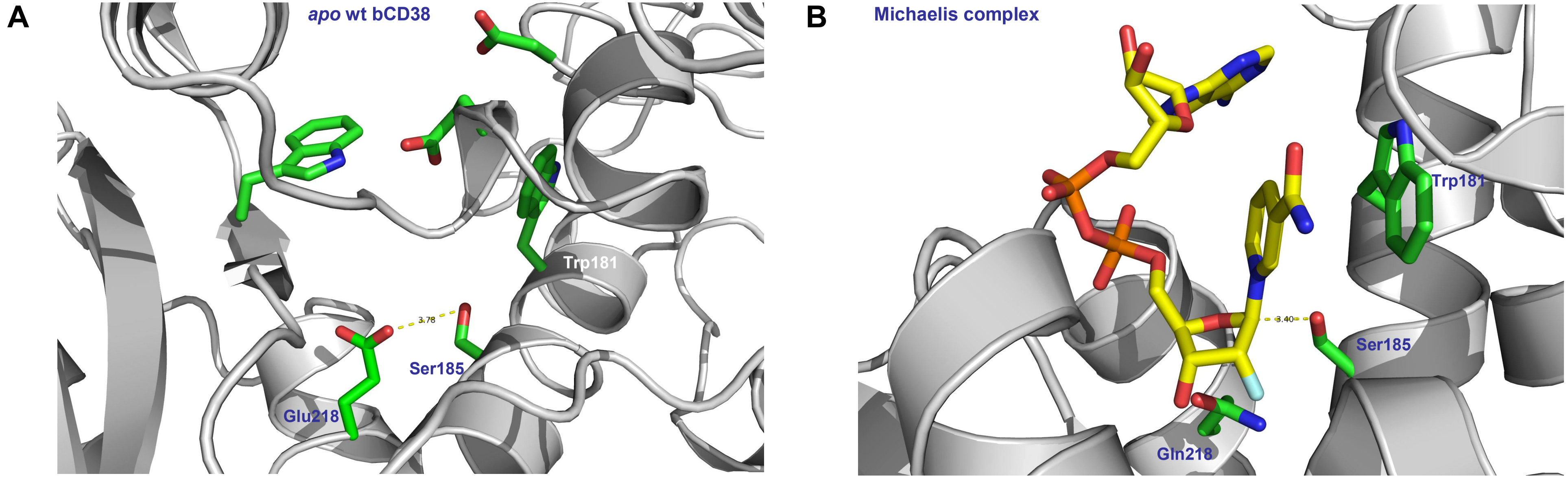

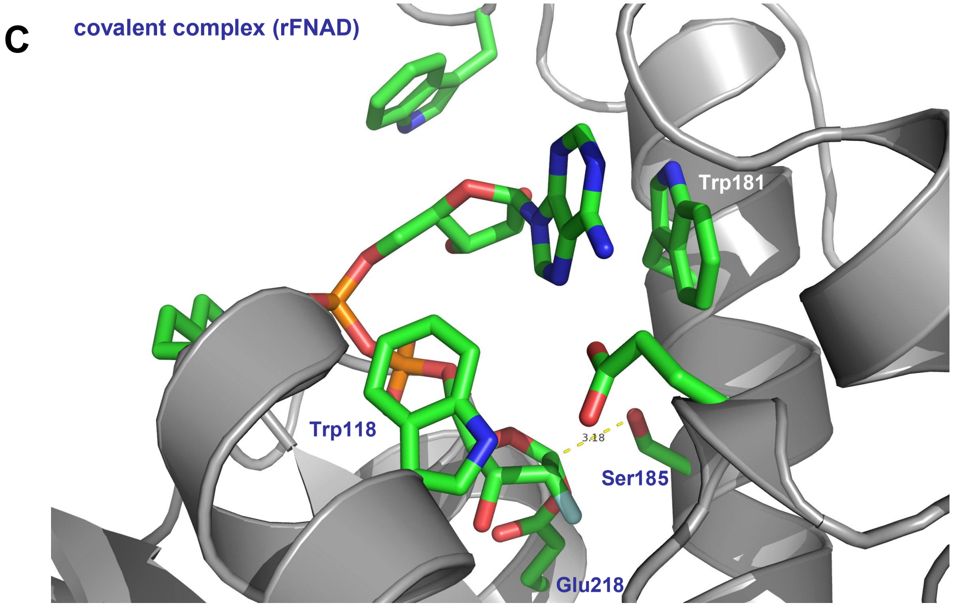

Supporting Information

Fig. S8 Positioning of Ser185 relative to Glu/Gln218 and to ribosyl C1'. In apo wt bCD38 the distance between the Oε2 atom of Glu218 and the side chain oxygen atom of Ser185 is 3.7-3.8 Å (**A**). The distance between the side chain oxygen atom of Ser185 and ribosyl C1' is 3.4 Å in the rFNAD/E218Q bCD38 Michaelis complex (**B**) and 3.2 Å in the covalent complex obtained by reaction of wt bCD38 with rFNAD (**C**).
