# Supplementary material for: Insights into the Mechanism of Bovine CD38/NAD+Glycohydrolase from the X-Ray Structures of Its Michaelis Complex and Covalently-Trapped Intermediates
Source: PLoS One. 2012 Apr 18;7(4):e34918. doi: 10.1371/journal.pone.0034918 (PMC3329556; doi:10.1371/journal.pone.0034918)
Supplement: Supporting Information S1 — Nature of the interactions between nicotinamide and Trp181 indole ring in bCD38. (PDF) [file pone.0034918.s010.pdf]

Supporting Information

Supplementary Data

Nature of the interactions between nicotinamide and Trp181 indole ring in bCD38

Prediction of the position of free nicotinamide in the active site of bCD38 was based on the X-ray complex between nicotinamide and human CD38 (PDB: 3dzg) [1], and then compared to an ideal positioning of free nicotinamide in stacking interaction with an indole ring. The modelling study described below suggests that neither the free nicotinamide nor the nicotinamide moiety of rFNAD can make an efficient π-π stacking with Trp181 side chain. In rFNAD, the nicotinamide is pulled to the bottom of the enzyme active site, partly due to strong hydrogen bonding to Glu138. Upon cleavage of the scissile bond, the nicotinamide moiety is slightly pushed out of the site, by forming a strong hydrogen bond with Asp147. In turn, the hydrogen bond to Glu138 is weakened. A further repositioning is necessary to optimize the stacking of nicotinamide to Trp181 indole. This and the concomitant vanishing of the hydrogen bonds with Glu138 and Asp147 could help nicotinamide to get finally out of the cavity.

Methods

1. Threading through the human complex

The pose of free nicotinamide into the active site of bCD38 was modelled based on the crystal structure of free nicotinamide bound to human CD38 (hereafter called *NCA_free*). The 3D-structure of the four amino acids which interact with the nicotinamide ring (i.e., Trp118, Glu138, Asp147 and Trp181 in bovine CD38) is common to the human and bovine enzymes, with RSMD of 0.67Å for the best-fit superposition of their side chains in 3ghh (bCD38) and 3dzg (hCD38) structures. To model the nicotinamide bound to the bovine enzyme, the B-chain of 3ghh was overlaid to the A-chain of 3dzg for the match of the indole atoms in bCD38 Trp181 and hCD38 Trp189.

2. Molecular mechanics on a simplified system

Both 3ghh B-chain and 3dzg A-chain were processed using MOE2009.10 to predict the pose of free nicotinamide with respect to a indole ring (hereafter called *NCA_opt*). Nicotinamide and Trp181/Trp189 side chains were respectively extracted from the ligand and bovine /human enzymes. Partial charges were assigned to both molecules using the Gasteiger method [2]. Hydrogen atoms were added to fill free valences. The system was subjected to energy

minimization to a rms gradient of 0.001 kcal/mol/Å on the MMFF94x energy surface (default MOE parameters, except the solvation energy contributions which were computed using the Born method [3]. All atoms of the indole ring were constrained using a harmonic constant of 100 kcal/mol/Å. The minimizations starting from 3ghh and 3dzg data converged to a unique solution.

Results

The results are given in Table S1. In the complex between rFNAD and bCD38, the nicotinamide (hereafter called NCA_rFNAD) establishes an efficient hydrogen bond with the glutamate residue of the "signature motif" (Glu138), and a weak hydrogen bond with Asp147. Its ring is parallel to the indole plane of the conserved Trp181. In the modelled complex between free nicotinamide and bCD38, the strong hydrogen bond is weakened and conversely the weak hydrogen bond is strengthened. This results from a tilt of the nicotinamide plane with respect to the indole ring, and a shift of the free nicotinamide away from the bottom of the bCD38 active site pocket (the amplitude of motion is about 1Å, as evaluated by the distance between the N1 coordinates in NCA_rFNAD and NCA_free). The force field optimized positioning of a free nicotinamide in interaction with an indole ring is again different, with a significant twist (the rotation around an axis centred at the pyridine centroïd and perpendicular the pyridine plane is about 50°), which forces the amide moiety of NCA_opt to move towards the outside of the enzyme active site, thereby abolishing the possibility to make hydrogen bonds with Glu138 and Asp147.

Table S1. Interaction between nicotinamide and bCD38.

Nicotinamide pose	π-π stacking [a]			H-bonds [b]	
	distance (Å) to the indole centroïd		Angle[c] (°)	distance (Å) to	
	NCA(N1)	NCA(C_{amide})		Asp147 Oδ	Glu138 Oε
NCA_rFNAD [d]	**5.25**	3.81	2.1	3.30	**2.67**
NCA_free	4.26	3.78	**15.1**	**2.72**	2.93
NCA_opt	4.32	**4.33**	2.5	**3.87**	**5.51**

[a] Evaluation of π-π stacking between Trp181 and NCA rings
[b] Evaluation of hydrogen bonds between NCA (amide) and CD38 ("signature motif")
[c] Angle between indole and nicotinamide rings
[d] Nicotinamide in the rFNAD/bCD38 Michaelis complex

References

1. Liu Q, Kriksunov IA, Jiang H, Graeff R, Lin H, Lee H C, Hao, Q. (2008) Covalent and Noncovalent Intermediates of an NAD Utilizing Enzyme, Human CD38. Chem Biol 15: 1068-1078.
2. Gasteiger J, Marsili M (1980) Iterative partial equalization of orbital electronegativity - a rapid access to atomic charges. Tetrahedron 36: 3219-3228.
3. Labute P (2008) The generalized Born/volume integral implicit solvent model: estimation of the free energy of hydration using London dispersion instead of atomic surface area. J Comput Chem 29: 1693-1698.
